# Recent advances in nanomedicines for photodynamic therapy (PDT)-driven cancer immunotherapy

**DOI:** 10.7150/thno.67300

**Published:** 2022-01-01

**Authors:** Bin Ji, Minjie Wei, Bin Yang

**Affiliations:** 1 School of Pharmacy, China Medical University, Shenyang, Liaoning 110122, China.; 2 The First Affiliated Hospital, China Medical University, Shenyang, Liaoning 110001, China.

**Keywords:** cancer immunotherapy, immune response, photodynamic therapy, emerging nanomedicines, synergistic nanotherapeutics

## Abstract

Cancer immunotherapy has made tremendous clinical progress in advanced-stage malignancies. However, patients with various tumors exhibit a low response rate to immunotherapy because of a powerful immunosuppressive tumor microenvironment (TME) and insufficient immunogenicity of tumors. Photodynamic therapy (PDT) can not only directly kill tumor cells, but also elicit immunogenic cell death (ICD), providing antitumor immunity. Unfortunately, limitations from the inherent nature and complex TME significantly reduce the efficiency of PDT. Recently, smart nanomedicine-based strategies could subtly modulate the pharmacokinetics of therapeutic compounds and the TME to optimize both PDT and immunotherapy, resulting in an improved antitumor effect. Here, the emerging nanomedicines for PDT-driven cancer immunotherapy are reviewed, including hypoxia-reversed nanomedicines, nanosized metal-organic frameworks, and subcellular targeted nanoparticles (NPs). Moreover, we highlight the synergistic nanotherapeutics used to amplify immune responses combined with immunotherapy against tumors. Lastly, the challenges and future expectations in the field of PDT-driven cancer immunotherapy are discussed.

## 1. Introduction

Conventional antitumor therapies including surgical resection, chemotherapy, radiotherapy, and molecular targeted therapy are used to effectively treat the early-stage tumors, while they are still helpless for advanced-stage disease 1. Encouragingly, cancer immunotherapy could prevent the recurrence of cancer and prolong the survival times of end-stage patients by activating the hosts' immune system 2. So far, numerous immune-based therapies have been approved for cancer treatment, such as checkpoint blockade immunotherapy, adoptive cell therapy (ACT), and cancer vaccines 3-6. Among these therapies, immune checkpoint therapy has successfully shifted the clinical research landscape. For example, the first FDA approved checkpoint inhibitor, therapeutic antibody ipilimumab, was used to block cytotoxic T lymphocytes (CTL) A-4, leading to a prominent regression of metastatic melanoma 7. Additionally, treatment with PD-1 blocking antibodies (e.g., nivolumab) significantly improved the objective response rate in many advanced cancers 8.

Despite the aforementioned benefits of immune checkpoint therapy, a large number of patients with various cancers are not sensitive to immune checkpoint inhibitors due to their low tumor immunogenicity 9-11. When a photosensitizer (PS) absorbs a photon of light, it can experience the following three different fates: First, the PS is activated from the ground state to a short-lived excited singlet state, and then the excited PS may decay back to the ground state by emitting fluorescence. Second, the abovementioned short-lived excited singlet state of the PS may undergo intersystem crossing to form a relatively long-lived triplet state. The triplet excited PS can interact with some endogenous substances to form a free radical (e.g., H_2_O_2_ and O_2_·^-^). Alternatively, the relatively long-lived triplet state can directly interact with molecular oxygen to form ^1^O_2_. In most cases, the ROS generated by PS in PDT mainly refers to the last process 12, 13. Recently, PSs activated by local laser irradiation induced PDT and selectively damaged the tumor tissue instead of normal organs 14-17. Compared to surgery, PDT is less invasive. More importantly, several studies have confirmed that PDT also stimulated antitumor immune response through a variety of mechanisms 18. Some examples are as follows: 1) Calreticulin (CRT), located on the endoplasmic reticulum (ER), moves to the cell membrane's surface undergoing PDT and provides an “eat me” signal to cause an immune response 19. 2) Upregulation of transcription factor expression (NF-κB), protein (heat shock protein 70) 20, or promoting secretion of cytokines (IFN-γ, IFN-α) 21. 3) Promoting mutations of antigen presenting cells (APCs) and CTLs to homing 22. 4) Recruitment of neutrophils to kill cancer cells and then causing the infiltration of macrophages 23, 24. Among them, PS-generated PDT can excellently initiate an immunogenic cell death (ICD), which is the most commonly used methods to induce immune response. With the progress of ICD, tumor cells show sequential changes 24. For instance, CRT and heat shock proteins (HSPs) will be exposed on the surface of dying cells. Adenosine triphosphate (ATP) and high-mobility group box 1 protein (HMGB1) will be released from the dying cells. The released ATP drives the APCs into tumor bed. The exposure of CRT facilitates the incorporation of APCs to phagocytose dead cells. The released HMGB1 promotes the TAA presentation to APCs. The maturation and migration of DCs is mediated by the expression of HSPs. In addition, the ICD can further boost the APC maturation via secreting proinflammatory cytokines. Therefore, the ICD can significantly facilitate the activation of immune response. More importantly, the ICD effectively serves as a bridge to connect the PDT with immunotherapy for cancer treatment 24, 25.

However, several factors significantly limit the efficacy of PDT, which further reduces their capability to induce immune response. First, tumor hypoxia can weaken the efficacy of oxygen-dependent PDT, and the oxygen consumption of PDT will further aggravate tumor hypoxia, leading to a vicious circle 26. Second, most PSs are activated by a short-wavelength light (400-700 nm), significantly hindering PDT for the treatment of deep-seated tumors due to the inferior tissue penetration ability of specific light 27. Third, PSs in high concentrations usually cause aggregation-caused quenching (ACQ), which will deeply weaken the optical property of PSs 28. Fourth, systemic administration of PSs might cause phototoxicity due to off-target distribution and accumulation in normal tissues 29, 30.

**Figure 1:**
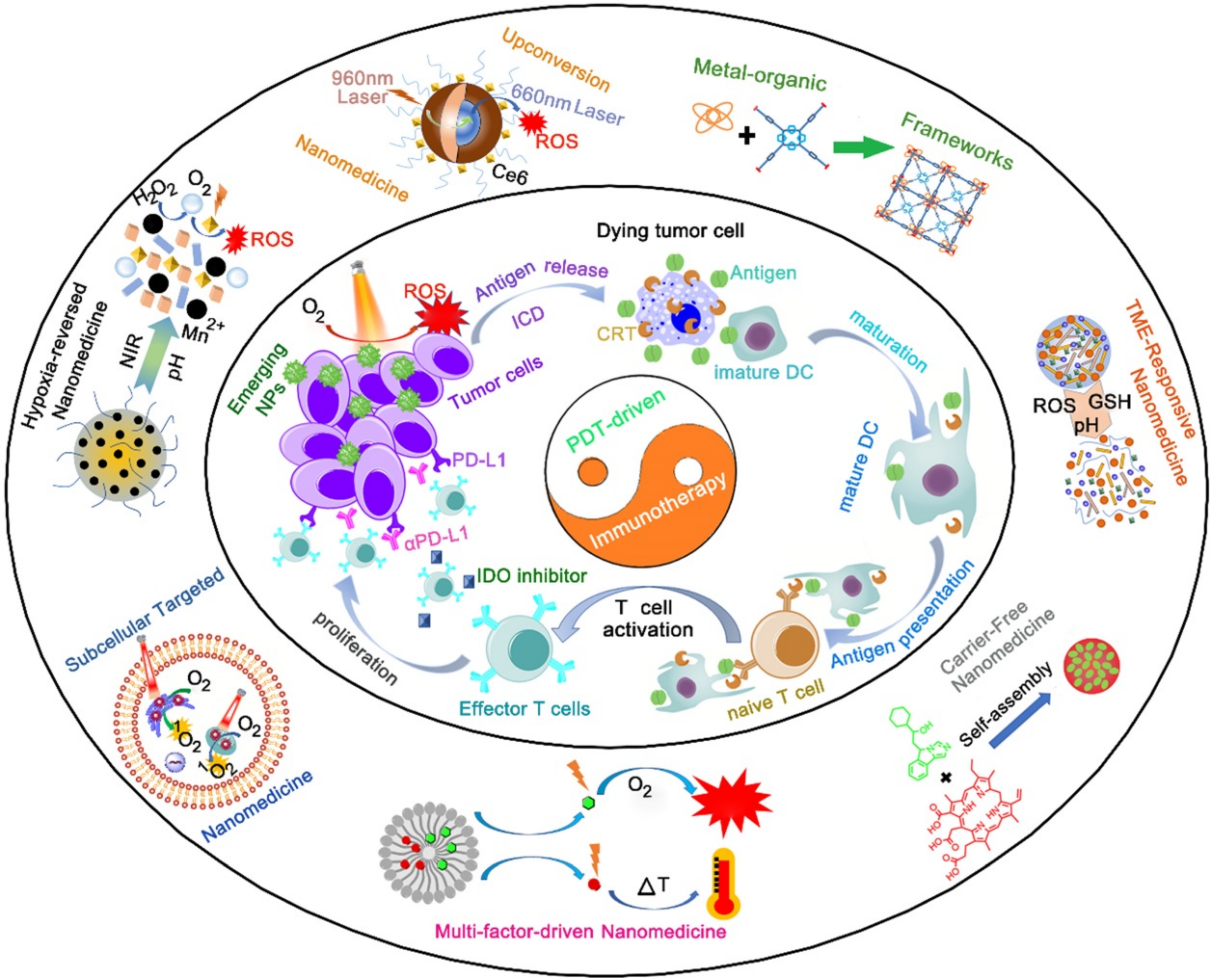
Schematic illustration of emerging nanomedicines for PDT-driven cancer immunotherapy. The outer shell shows recent nanomedicines developed for PDT-driven cancer immunotherapy, including tumor hypoxia-reversed nanomedicines, UCNs, nanosized nMOFs, TME-responsive NPs, subcellular targeted NPs, carrier-free and small-molecule prodrug-based self-assembled NPs, and multifactor-driven nanomedicines. The inner core explains how to amplify the combination of PDT and immunotherapy, thereby improving the effect of cancer treatment.

Fortunately, advanced nanomedicines have opened new promising avenues to refine and improve the performance of PDT, leading to an enhanced immune response 31. Advanced nanomedicines can solve the tumor hypoxia problem by combining various self-supply oxygen strategies. Using emerging nanocarriers, nanomedicines can control the *in vivo* distribution of PSs to achieve tumor-targeting accumulation. In addition, smart tumor microenvironment (TME)-responsive nanoplatforms not only reduce phototoxicity by avoiding the premature leakage of PS in blood circulation, but also accelerate the release of PSs to overcome the ACQ effect at the tumor site. More importantly, nanomedicines excellently codeliver PSs and immunomodulators to their targets, resulting in an optimal cancer immunotherapy. Herein, we review the emerging nanomedicines for PDT-driven cancer immunotherapy. Moreover, we highlight the synergistic nanotherapeutics for increasing immune responses combined with immunotherapy against tumors (**Figure 1**). Finally, the current challenges and future perspectives in the field of PDT-driven cancer immunotherapy are also highlighted.

## 2. Emerging nanomedicines for PDT-driven cancer immunotherapy

Conventional immunotherapies are usually unable to transform nonimmunogenic (cold) tumors into immunogenic (hot) tumors 32, 33. Encouragingly, nanomedicine-based therapeutic strategies have the capability to transform cold into hot tumors, especially for aforementioned PDT with several immunostimulatory mechanisms to initiate the immune response 34, 35. Recently, advanced nanomedicines have been designed to improve the efficacy of PDT, including tumor hypoxia-reversed nanomedicines, TME-responsive NPs, upconversion NPs (UCNs), and nanosized metal-organic frameworks (nMOFs). The optimized PDT combined with immunotherapy would achieve a strong synergistic effect against advanced cancers.

### 2.1. Tumor hypoxia-reversed nanomedicines for PDT-driven cancer immunotherapy

Rapid tumor growth causing insufficient blood supply and local oxygen consumption of PDT aggravates tumor hypoxia, seriously affecting the efficacy of PDT 36, 37. In addition, tumor hypoxia can suppress antitumor immunity by facilitating the proliferation of immunosuppressive cells (e.g., M2 type macrophages) to promote tumor development and recurrence 38-40. Hence, alleviating hypoxia in the tumor site is an important method to improve the efficacy of PDT-driven cancer immunotherapy. Recently, researchers developed various biomaterials and therapeutic agents to alleviate tumor hypoxia, including hemoglobin, catalase (CAT), manganese dioxide NPs, oxygen-shuttle nanoperfluorocarbon (nanoPFC), hyaluronidase (HAase), and metformin (Met) (**Table 1**) 41-46.

#### 2.1.1. H_2_O_2_-activated oxygen-producing NPs (HAOP NPs) for PDT-driven cancer immunotherapy

**Figure 2:**
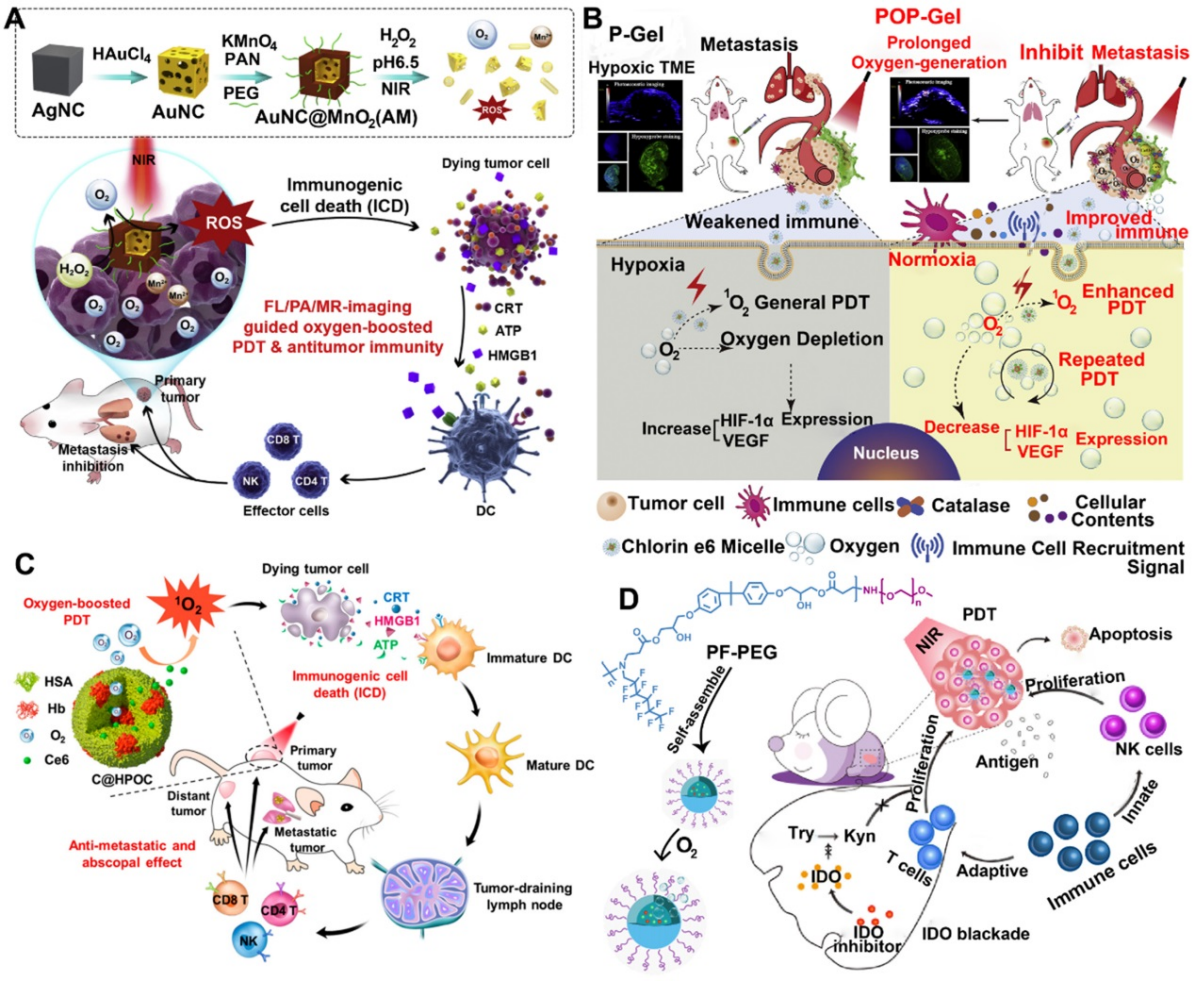
Schematic illustration of various tumor hypoxia-reversed nanomedicines for PDT-driven cancer immunotherapy. (A) AuNC@MnO_2_ (AM) NPs, (B) Oxygen-producing phototherapy hydrogel (POP-Gel), (C) Ce6@(Oxygen-carrying hybrid protein) NPs, (D) Oxygen-carrying fluorinated polymer NPs (FPNs). Reproduced with permission 41, 43, 44, 52. Copyright 2018, Elsevier Ltd; Copyright 2019, Elsevier Ltd; Copyright 2018, American Chemical Society; Copyright 2019, Elsevier Ltd.

Nowadays, some inorganic biomaterials (e.g., manganese dioxide (MnO_2_)) can activate hydrogen peroxide overexpressed in tumor cells to produce oxygen 47. Therefore, HAOP NPs can be used to solve the problem of tumor hypoxia. Furthermore, gold nanocages (AuNCs) mediated ROS production as intrinsic inorganic PSs, and gold-based nanomedicines can improve the PDT efficiency of other PSs owing to a localized electric field (LEF) 48, 49. Based on these theories, Liang et al. designed a TME-responsive core-shell AuNC@MnO_2_ (AM) nanoplatform for oxygen-boosted PDT combined with immunotherapy against metastatic breast cancer (**Figure 2A**) 41. Under acidic TME conditions, the MnO_2_ shell degrades and catalyzes the reaction of intracellular H_2_O_2_ to produce abundant oxygen, and then the self-generated O_2_ further improved the ROS production of AM upon light irradiation by ameliorating tumor hypoxia. More importantly, the oxygen-boosted PDT robustly induced more powerful ICD compared with the PDT caused by AuNCs, thereby enhancing the antitumor immune response. As a result, this oxygen-boosted AM nanoplatform not only effectively inhibited the local tumor growth but also prevented the tumor metastases 41, 50. However, gold-based nanomedicines cause some problems for the organism, including difficulty in degradation or excretion, a potential toxic effect 51. Fortunately, recent studies showed that biodegradable CaCO_3_/MnO_2_-based nanocarriers can be used to deliver therapeutic cargos and serve as contrast agents 42. Moreover, this type of nanocarriers can be responsive to TME and modulate it. For instance, Liu et al. designed a TME-responsive biodegradable nanoplatform by combining PDT and immune checkpoint therapy for improving cancer treatment. This biodegradable nanoplatform (Mn@CaCO_3_/ICG@siRNA) was constructed from MnO_2_ NPs covered with an ICG-doped acid-sensitive layer of CaCO_3_ and loaded with PD-L1-targeting siRNA through electrostatic interaction 42. After reaching the acidic TME, the layer of CaCO_3_ was rapidly decomposed into CO_2_ and Ca^2+^ (biological components), and subsequently the core of MnO_2_ NPs triggered the reaction of tumor intracellular H_2_O_2_ to produce H_2_O and O_2_, which not only reversed the tumor hypoxia but also provided the necessary elements of PDT (O_2_). Compared with free ICG, Mn@CaCO_3_/ICG@siRNA produced much more ^1^O_2_ owing to the MnO_2_-mediated HAOP NPs, thereby significantly improving the efficacy of PDT. More importantly, a combination of PDT and siRNA provided synergistic benefits by enhancing the tumor immunogenicity via PDT and silencing the PD-L1 gene by siRNA, thus leading to a powerful antitumor efficiency.

In addition to MnO_2_ NPs, CAT, an endogenous antioxidant enzyme, also efficiently triggered the decomposition of tumor intracellular H_2_O_2_ to generate O_2_ for relieving tumor hypoxia, thus increasing the anticancer efficacy of PDT and remodeling the immunosuppressive TME 52, 53. For instance, Jiang et al. developed an oxygen-producing phototherapy hydrogel (POP-Gel) to inhibit the proliferation and metastasis of breast cancer (**Figure 2B**). In this study, they fabricated hydrogel scaffolds using FDA-approved Pluronic^®^ F127 and F68, which is loaded with Ce6 as the PS and CAT assisted by CaO_2_ as a catalyst to continuously supply oxygen for a long time to alleviate tumor hypoxia 52. With immunofluorescence staining and photoacoustic (PA) imaging, they showed that the oxygen generated using this platform could be prolonged for up to five days, thereby realizing the strategy (“once injection, sustained treatment”). As a result, the hypoxic regions of tumors were reduced, thus significantly improving the PDT efficacy and decreasing the expression of hypoxia-related factors. Furthermore, the improved PDT induced an extreme immune response, enhancing the inhibition of cancer growth.

Although the oxygen-producing phototherapy nanoplatform effectively enhanced the post-PDT-triggered immune response, the immunosuppressive TME hindered the ultimate efficacy of PDT-mediated cancer immunotherapy by suppressing the activation of CTLs 54. To overcome these hurdles, Liu et al. reported a synergistic treatment strategy by utilizing oxygen-boosted PDT combined with checkpoint blockade therapy for eliminating metastatic tumors 54. First, they designed a light-triggered hydrogel composed of Ce6 modified CAT, biodegradable polymer (poly(ethylene glycol) double acrylate, termed as PEGDA), and immune adjuvant (R837-loaded PLGA NPs). After the intratumor injection of precursor solution upon laser irradiation, the polymeric matrix helped in the polymerization of PEGDA by ROS to form *in situ* gelation, thus retaining various agents (Ce6-CAT and immune adjuvant). Subsequently, Ce6-CAT by repeated laser stimulation continuously generated ROS to induce strong ICD and regulate the polarization of macrophage, as the retained CAT could overcome tumor hypoxia by persistently producing O_2_. Furthermore, the PDT-mediated ICD, along with the immune adjuvant, would induce a significantly enhanced immune response 54. More importantly, application of α-CTLA4 blockade therapy significantly suppressed the activation of Treg, further improving the multiround PDT-mediated immunotherapy.

#### 2.1.2. Oxygen-carrying NPs for PDT-driven cancer immunotherapy

In hypoxic solid tumors, the efficacy of PDT is largely reduced by hypoxia. To overcome this challenge, many efforts have been made to decompose H_2_O_2_ to supply O_2_. However, the H_2_O_2_ levels in some parts of the tumor are very low, and the results are significantly limited 55, 56. Fortunately, some studies have shown that hemoglobin, red blood cells, and PFC carry O_2_ directly to hypoxic tumors 43, 44. Chen et al. designed a hybrid protein oxygen nanocarrier with Ce6 for oxygen self-sufficient PDT and achieved cancer immunotherapy (**Figure 2C**) 43. This hybrid protein oxygen nanocarrier is composed of hemoglobin (Hb) and human serum albumin (HSA). This is because the excellent oxygen-transport carrier (free Hb) has a short circulation time and poor stability, while HSA exhibits good* in vivo* stability and biocompatibility 43. This hybrid protein oxygen nanocarrier can significantly reduce tumor hypoxia by simultaneously codelivering the PS and oxygen to the tumor, thereby significantly improving the effect of PDT and the infiltration of CD8^+^ T cells at the tumor site. More importantly, the increased PDT induced a strong ICD by improving the release amount of damage-associated molecular patterns (DAMPs) to activate more dendritic cells (DCs), NK cells, and CTLs, thus effectively inhibiting the primary tumors and suppressing lung metastasis 43. Similarly, a recent study developed auxiliary liposomes loaded with oxygen-laden Hb to enhance the efficacy of PDT caused by ICG modified gold nanospheres 57. After the liposomes loaded with oxygen-laden Hb reached the tumor hypoxia condition, the oxygen was rapidly released from the Hb, assisting the ICG to generate robust ROS, thus improving the intensity of immune response.

Aside from Hb, PFCs can store O_2_ molecules like a reservoir, as PFCs have weak intermolecular cohesive forces to boost the insertion of oxygen molecules 58. Xing et al. developed a versatile nanosystem consisting of fluorinated polymer NPs (FPNs) with the simultaneous encapsulation of NLG919 and Ce6 for improving the efficacy of PDT and modulating immunosuppressive TME (**Figure 2D**) 44. Compared to alkylated polymer NPs, FPNs showed high oxygen carrying capacity, thereby generating much more ROS and treating hypoxic tumor. Meanwhile, the enhanced PDT and remodeled TME also induced increased immunological responses and promoted the proliferation of CTLs. More importantly, a combination of indoleamine 2,3-dioxygenase (IDO) blockade therapy and PDT achieved more effective antitumor results compared with PDT alone, as the encapsulated NLG919 inhibited the IDO pathway, thus restoring the activation of CTLs 44.

#### 2.1.3. ECM-degraded and respiration-inhibited NPs for PDT-driven cancer immunotherapy

The TME contains an abnormally condensed extracellular matrix (ECM), which not only reduces the oxygen supply via squeezing the tumor blood vessels but also impedes the diffusion of oxygen by serving as a physical barrier 59-61. The dense ECM is composed of some glycosaminoglycan, proteins, etc., among which the cross-linked hyaluronic acid (HA) plays an important role in forming the physical barrier 45, 61. HAase is a specifical enzyme to hydrolyze HA. Hence, the application of HAase to decompose HA is a promising strategy to relieve tumor hypoxia 45. For instance, Wang et al. designed a cascade delivery strategy for improving the TME and enhancing PDT-mediated cancer immunotherapy 45. First, they developed a natural biocompatible polymer nanoparticle system via the self-assembly of dextran-modified HAase (DEX-HAase) by inserting pH-responsive linkers. Compared to free HAase, the DEX-HAase NPs significantly improved the stability of HAase in blood circulation. Furthermore, because of the presence of pH-responsive linkers, the DEX-HAase NPs selectively triggered the release of HAase to decompose the HA after accumulating in the acidic TME, loosening the condensed ECM 45. The loosened tumor ECM further increased the penetration of oxygen molecules to relieve tumor hypoxia and reverse the immunosuppressive TME. After the first wave injection of DEX-HAase NPs, the remodeled TME enhanced the efficacy of PDT caused by the second wave injection of Ce6@liposome plus irradiation 45. More importantly, the enhanced PDT induced an intense immune response, and the PDT-mediated cancer immunotherapy was further improved by combining with PD-L1 blockade therapy after injecting the third wave of anti-PD-L1. As a result, this sequential injection of therapeutic agents achieved cooperative PDT-immunotherapy, effectively inhibiting the growth of primary tumor as well as distant tumor 45.

Apart from anti-PD-L1, clustered regularly interspaced short palindromic repeats associated protein 9 (CRISPR-Cas9) could effectively damage the protein tyrosine phosphatase nonreceptor type 2 (Ptpn2) gene, resulting in the enhanced activation of CD8^+^ T cells. For example, Qian et al. designed a core-shell nanosystem to achieve a combined photodynamic-immunotherapy, in which the core is composed of modified PSs and Cas9-Ptpn2 plasmids 62. With the administration of HAase in advance, both the HA shell of the nanosystem and the HA shell in tumor ECM were degraded, thus improving the penetration of loading cargos and O_2_. Therefore, this nanosystem under laser irradiation could trigger an enhanced PDT because of the alleviated hypoxia to induce a strong immune response. Furthermore, the Cas9-Ptpn2 plasmids in this nanosystem were developed to damage the Ptpn2 gene, leading to increasing proliferation of T lymphocytes and secretion of cytokines (IFN-γ, IFN-α) 62.

The inhibition of respiration could decrease the oxygen consumption of tumor cells, which offers an indirect way to enhance the supply of oxygen for PDT. Recently, many studies reported that Met could decrease oxygen consumption by inhibiting mitochondrial respiratory chain 46, 63. For example, Teng et al. constructed a platelet-mimicking nanoparticle system composed of Met and PS (IR780) to integrate immunosuppressive reversion and immunogenic activation. In this study, IR780 could initiate an oxygen-boosted PDT with the help of Met-mediated mitochondrial respiration inhibition, resulting in an ICD-based immunogenic activation. Moreover, the alleviated tumor hypoxia by reducing oxygen consumption weakened the immunosuppression mediated by myeloid-derived suppressor cells (MDSC) 46. In another study, Shen et al. developed a multifunctional liposome coloaded with IR775 and Met. They found that Met not only reversed tumor hypoxia to enhance IR775-induced PDT, but also regulated the endoplasmic-reticulum-associated degradation by pAMPK pathway to reduce the PD-L1 expression 63.

### 2.2. UCNs for PDT-driven cancer immunotherapy

Most PSs for PDT are activated by a short wavelength (e.g., visible light), leading to a limited penetration depth in live tissues. Furthermore, the chromosomes (hemoglobin, etc.) of organization can strongly absorb visible light, significantly interfering with the PS on the transformation of light 64-66. Therefore, the efficacy of PDT with the depth of tissue significantly reduced, resulting in incomplete treatment and recurrence. To find an effective method to overcome the depth of tissue penetration has become an urgent need to solve the problems of PDT at this stage.

UCNs are nanometer-sized materials that convert low-energy light to high-energy light through sequential excitation with multiple photons via an anti-Stokes emission process. Compared with downconverted NPs, UCNs can absorb near-infrared (NIR) light and have a relatively high depth of tissue penetration, while the light can be converted into strong UV or visible light 12, 66. Based on this feature, UCN-based PDTs have been extensively studied for tumor therapy to improve the tissue penetration depth. For example, Ai et al. constructed Ce6-loaded UCNs combined with HA plus MnO_2_ nanosheets to improve NIR light-mediated PDT 67. The Ce6-loaded UCNs could be efficiently activated to generate sufficient ^1^O_2_ for inducing both deep-tissue cellular ablation by converting 808 nm light excitation into 655 nm emissions. Furthermore, the surface-anchored HA significantly inhibited the tumor recurrence post-PDT treatment by producing M1-type macrophages instead of M2-type macrophages.

**Figure 3:**
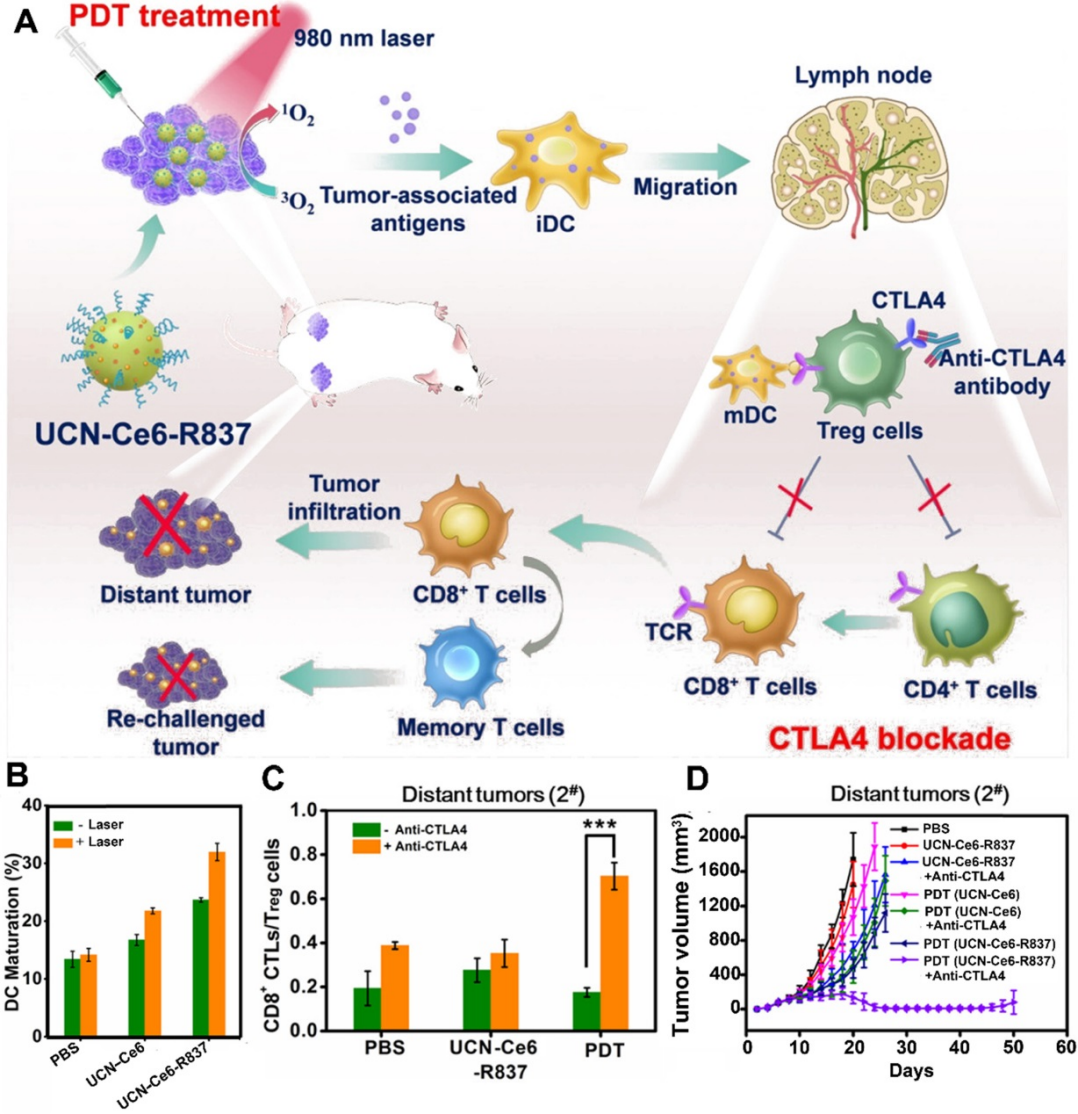
Upconversion NPs for PDT-driven cancer immunotherapy. (A) Schematic illustration of UCN-Ce6-R837 nanoplatform-induced strong immune response, which combined with CTLA4 blockade to inhibit the relapse and metastasis of tumors. (B) DC maturation induced by various formulations. (C) Frequency of CD8^+^ CTLs\Treg cells in distant tumors treated with different formulations. (D) Growth curves of CT26 tumor-bearing mice after receiving different formulations. Reproduced with permission 68. Copyright 2017, American Chemical Society.

In addition to M1-type macrophages, the CD8^+^ T cells also play an important role in preventing tumor recurrence. However, the activation of CD8^+^ T cells was suppressed by regulatory T cells (T_reg_) 68. Fortunately, several studies have reported that the activity of T_reg_ cells was effectively inhibited by checkpoint blockade (e.g., anti-CTLA-4 antibody) 68, 69. For instance, Xu et al. designed a multitasking nanoplatform composed of UCNs as an NIR-responsive vehicle, imiquimod (R837) as an immune adjuvant, and Ce6 as a PS (**Figure 3**) 68. The UCN-based platform overcame limited penetration depth by the application of NIR irradiation, thus triggering the loaded Ce6 to generate ROS. Subsequently, the generated ROS further destructed the tumor cells to produce numerous tumor-associated antigens (TAAs) accompanied by the loaded immune adjuvant (R837) to provide powerful immune responses. More importantly, the powerful immune responses caused by UCN-Ce6-R837-based PDT was further improved by using anti-CTLA-4 antibodies, which suppress the vitality of Treg cells, thus restoring the activity of CTLs. As a result, the abovementioned synergistic treatment strategy not only eliminated the distant tumors, but also showed prolonged immune memory effect and inhibited the tumor recurrence.

### 2.3. Nanosized nMOFs for PDT-driven cancer immunotherapy

During PDT, a high concentration of PSs in a tight core of NPs often causes ACQ effect, causing a reduced yield of ROS and self-quenching fluorescence 70, 71. Unlike the PS-loaded nanomedicines, nMOFs based on the incorporation of PSs as construction units have tunable and porous structures that overcome the ACQ effect and endow high PS loadings 70, 72. Furthermore, the porous structures of nMOFs can not only well disperse the PSs, but also facilitate the diffusion of ROS, thereby enhancing the efficacy of PDT 70. For instance, Cai et al. designed *in situ* tumor vaccines, consisting of MOF-based NPs (PCNs), immunologic adjuvant (CpG), and hypoxia-inducible factor inhibitor (acriflavine) 73. To achieve excellent tumor-targeting capacity, they coated the surface of PCN-CpG-acriflavine with HA to recognize the CD44 receptor overexpressed on tumor cell surface. In their study, the self-assembled PCNs were composed of zirconium ions and H_2_TCPP, and the well-dispersed H_2_TCPP in its framework induced a comparable PDT by overcoming the ACQ effect. Furthermore, the PCNs loaded with acriflavine suppressed the enhanced hypoxic signaling caused by PDT, thereby reversing the tumor hypoxia. More importantly, the PDT-generated TAAs combined with CpG achieved a synergistic effect to provide intensive immune responses 73. To further improve the immune response, Zhang et al. developed a fusion cytomembrane (FM)-coated nMOF for photoactivatable cancer immunotherapy, where the FMs were derived from DCs and tumor cells 74. In their study, the FMs not only showed an excellent tumor-targeting capacity because of the self-targeting characteristic to homologous tumors, but also facilitated tumor specific immunoresponses because of the presence of highly expressed tumor antigens. In addition, the DC derived immunomodulatory molecules in FMs both enhanced the antigen presentation and supported antigen-specific T cell response. As a result, the nMOF-mediated PDT combined with FM-induced immunotherapy provided strong immune responses 74.

**Figure 4:**
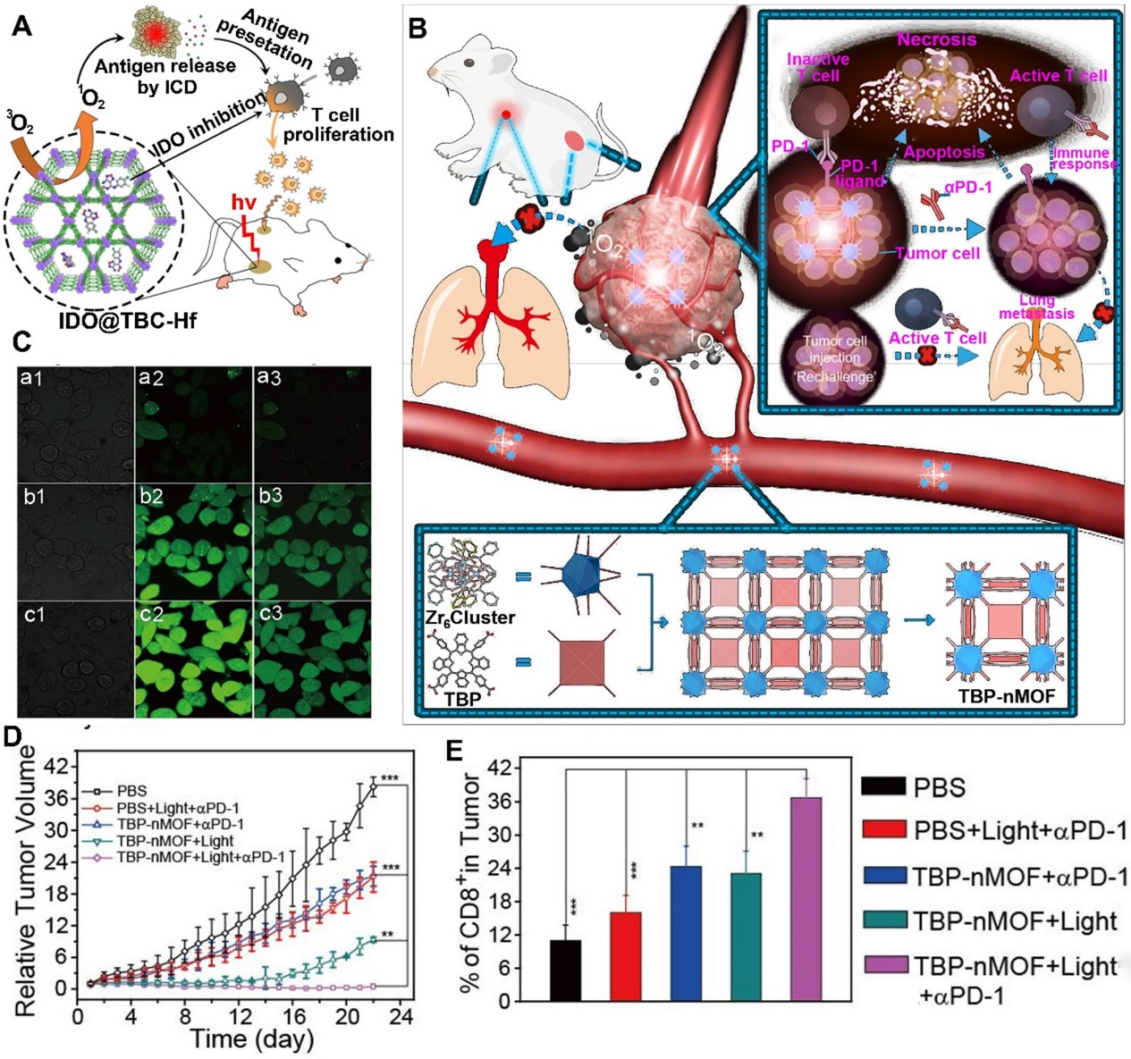
nMOFs for PDT-driven Cancer Immunotherapy. Mechanism of the nMOFs to induce a PDT-mediated immune response, and combination with (A) IDO inhibitor or (B) αPD-1 in cancer immunotherapy. (C) CLSM imaging of tumor cells incubated with DCFH-DA and TBP-nMOFs in (b) under dark, (c) 5% O_2_ or (d) 21% O_2_ under light irradiation. (D) Growth curves of 4T1 tumor-bearing mice after receiving different formulations. (E) Frequency of CD8^+^ cells in tumors treated with different formulations. Reproduced with permission 75, 76. Copyright 2016 and 2018, American Chemical Society.

Although the immune response activates the CTLs to kill cancer cells, the immunosuppressive TME suppressed the activation of CTLs via many negative immune mechanisms 77, 78. To restore the function of CTLs, several studies recently reported that the application of checkpoint blockade immunotherapy, such as IDO inhibitor, relieved the IDO-mediated immunosuppression 79. Moreover, the abovementioned MOFs could be efficiently loaded with IDO inhibitors in their open channels owing to the high coloading capacity. For instance, Lin et al. constructed H_4_TBC-based nMOFs and used those to encapsulate IDO inhibitors in their highly porous structure for inducing systemic antitumor immunity (**Figure 4A**) 75. They showed that the H_4_TBC-based nMOFs provided PDT and ICD; further combination with checkpoint blockade therapy using IDO inhibitor enhanced T cell infiltration in TME. As a result, this synergistic treatment strategies inhibited the growth of local and distant tumors.

In addition to the IDO inhibitor, antibodies (e.g., αPD-1 and αPD-L1) are another widely used immune therapeutic agents for checkpoint blockade strategies. For example, Zhang et al. developed benzoporphyrin-based nMOFs consisting of a Zr_6_ cluster and PS (TBP) in combination with αPD-1 to inhibit tumor metastasis (**Figure 4B-E**) 76. The benzoporphyrin-based nMOFs showed an enhanced ^1^O_2_ generation because of their π-extended TBP-based linkers, thus producing O_2_-dependent PDT even under hypoxic tumor condition 76. Moreover, the TBP effectively avoided ACQ because of its good dispersion in the structure of nMOFs, further facilitating PDT. Based on these advantages, the TBP-mediated PDT induced a strong ICD to recruit tumor infiltrating CTLs 76. More importantly, the combined application of αPD-1 restored the activity of CTLs suppressed in the immunosuppressive TME 76. In another study, Lin et al. constructed a cationic nMOF (W-TBP) highly loaded with anionic CpG via electrostatic interaction to improve cancer immunotherapy 80. The W-TBP upon laser irradiation provided PDT, thus enhancing the release of TAAs. In addition, compared to Bi(NO_3_)_3_·5H_2_O-based negative nMOF, the WCl_6_-based cationic nMOF showed a four-fold CpG loading capacity, which efficiently enhanced DC maturation, thereby further promoting the availability of antigen by DCs. Additionally, the αPD-1 combined with antigen availability significantly enhanced the infiltration and activation of CTLs in bilateral tumors, thus achieving a strong cancer immunotherapy 80.

### 2.4. TME-responsive NPs for PDT-driven cancer immunotherapy

Compared with normal tissues, solid tumors exhibit some characteristics of TME, including a low pH, severe hypoxia, and a high level of glutathione (GSH). Hence, the development of smart stimulus-responsive nanomedicines containing TME-sensitive chemical linkers or elements can regulate the release of their cargos. In addition to advanced nMOFs, the TME-responsive NPs can also effectively avoid the ACQ by cleaving the TME-responsive linkers to rapidly release PSs at the tumor site, thus significantly improving the yield of ROS 81. Meanwhile, the increased ROS will promote the ICD effect, thereby enhancing the immune response. Moreover, the TME-responsive NPs smartly program the location and pharmacokinetics of both PSs and immunomodulators to improve the tumor-targeting capability, leading to an enhanced PDT-driven cancer immunotherapy without causing severe side effects (**Table 2**).

#### 2.4.1. pH-responsive NPs for PDT-driven cancer immunotherapy

**Figure 5:**
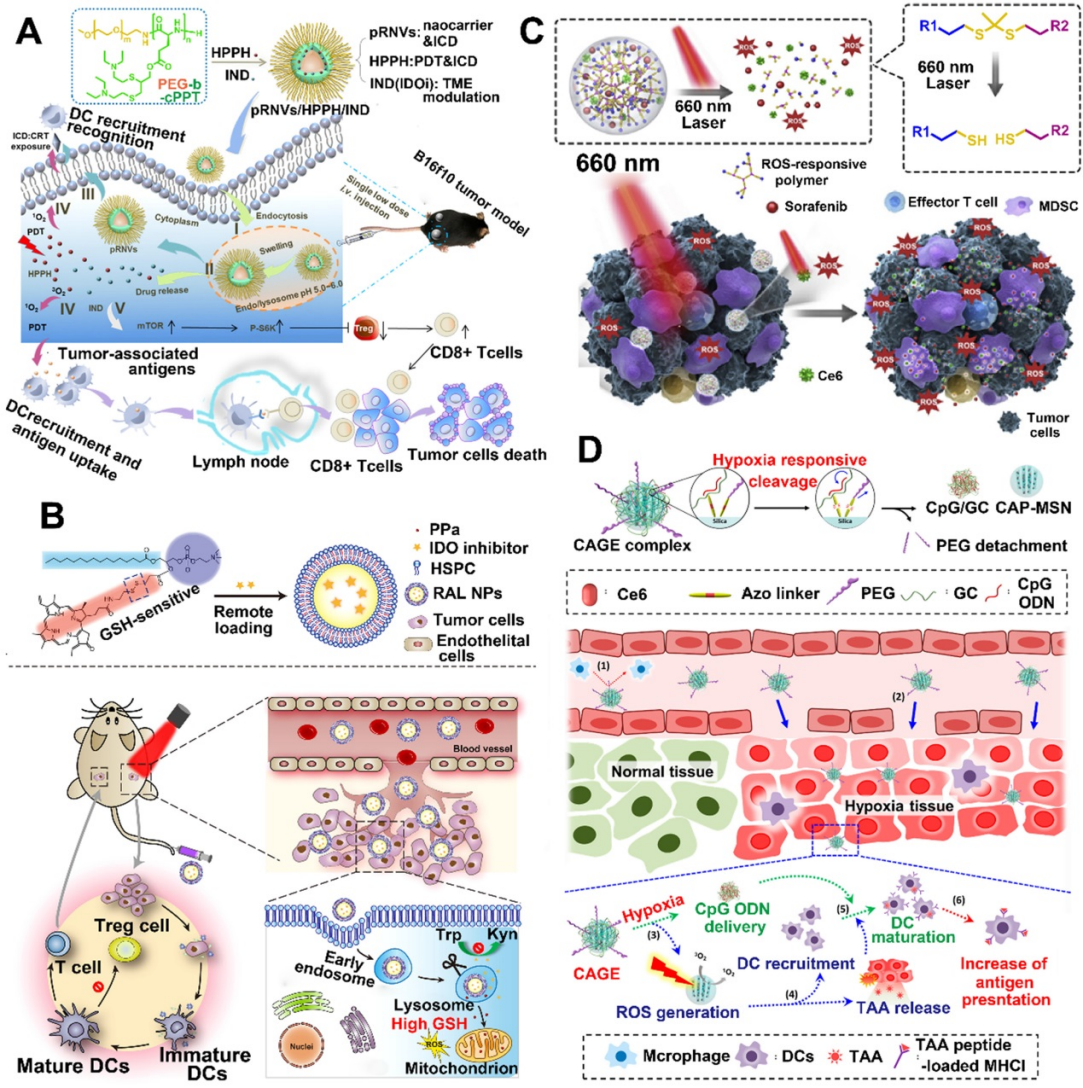
TME-responsive NPs for PDT-driven cancer immunotherapy. (A) Schematic illustration of pH-responsive NPs for high efficient PDT, induction of ICD, and inhibition of IDO pathway. Reproduced with permission 36. Copyright 2020, American Chemical Society. (B) Schematic illustration of GSH-responsive NPs for PDT-induced immune response and improved tumor-targeting cancer immunotherapy. Reproduced with permission 82. Copyright 2019, American Chemical Society. (C) Schematic illustration of ROS-responsive NPs for cascaded amplification of PDT and strong systemic antitumor immune responses. Reproduced with permission 83. Copyright 2020, Elsevier Ltd. (D) Schematic illustration of Hypoxia-responsive NPs for hypoxia-triggered conversion, PDT-induced immune response and vaccine immunotherapy. Reproduced with permission 84. Copyright 2019, American Chemical Society.

So far, pH-responsive NPs are very common; compared to the physiological pH of 7.4 in the blood and normal tissues, more acidic environments have been found in tumors 85. Wang et al. developed an acid-activatable multifunctional nanoplatform for PDT-mediated cancer immunotherapy 86. This nanoplatform is composed of pH-sensitive diblock copolymer (PDPA), pheophorbide A (PPa) which is conjugated to PDPA, and PD-1-PD-L1 interaction inhibitor (siRNA). The modified diblock copolymer (termed as PDPA-PPa) was self-assembled into micelles, and the apparent pKa of PDPA is about 6.3 86. As the PPa was compressed into a tight hydrophobic core, this nanoplatform during blood circulation decreased the phototoxicity due to the ACQ effect. After the nanoplatform was delivered to the weakly acidified TME, the rapid dissociation of micelles caused by PDPA's "proton sponge effect" restored the photoactivity of PPa. Afterwards, the activated PPa upon irradiation generated ROS by PDT and then stimulated the expression of NF-κB and HSP70 to provide immune response 86. Compared to PDT alone, PDT-induced immune response combined with blocking PD-L1 pathway by using siRNA significantly enhanced the proliferation of lymphocytes and cytokine secretion, leading to a long-lasting immune response. As a result, lung metastasis was suppressed by the immune memory response 86. Another work, Yang et al. designed pH-responsive nanovesicles (pRNVs) and coencapsulated PSs (HPPH) and IDO inhibitor (indoximod) for photodynamic immunotherapy, in which the polyethylene glycol-b-cationic polypeptide was self-assembled into pRNVs (**Figure 5A**) 36. Compared with the pRNVs encapsulated with HPPH and IND (pRNVs/HPPH/IND) in pH 7.4 after 24 h incubation, the pRNVs/HPPH/IND in pH 5.0 showed a triple drug release rate owing to the smart nanocarriers with an excellent swelling capacity in acidic condition. Thus, the specific release of HPPH upon irradiation not only produced singlet oxygen to directly kill tumor cells by PDT, but also provided an immune response by ICD effect 87. Surprisingly, the pRNVs also induced ICD to further improve the host immunity. More importantly, the simultaneously released IND, an IDO blockade, relieved tumor immunosuppression by restoring the mTOR pathway, thus enhancing the proliferation of CTLs 87.

#### 2.4.2. GSH-responsive NPs for PDT-driven cancer immunotherapy

Redox potential is another possible internal stimulus for responsive release, because the concentration of GSH inside cells is almost three orders of magnitude higher than that in the extracellular plasma. Moreover, the GSH level in tumor tissues is four-fold higher than that in normal tissues 88. Wang et al. developed a redox-activated liposome self-assembled by a porphyrin-phospholipid conjugate to synergistically work with IDO inhibitor to produce tumor immune response and reverse tumor immunosuppression (**Figure 5B**) 82. The authors found that the porphyrin-phospholipid conjugate induced ICD by PDT after a high concentration of tumor intracellular GSH triggered the cleavage of redox-sensitive linker, while the redox-activated liposome remained silent for the photoactivity during the blood circulation period 89. However, the ICD caused by PDT was significantly limited owing to the immunosuppressive IDO pathway. To solve this problem, IDO inhibitor (NLG-8189) was coencapsulated in a redox-activated liposome 90, 91. It could be released by triggering the disulfide bond cleavage in tumor site to reverse the immunosuppressive TME 92. As a result, this combination therapy of PDT-induced immunotherapy with IDO inhibitor not only suppressed the primary and abscopal tumors, but also inhibited the metastasis of breast cancer.

To avoid the leakage of encapsulated IDO inhibitor in blood circulation, Hu et al. designed a reduction-labile heterodimer of IDO inhibitor (NLG919) and PPa to achieve controllable drug release 93. Moreover, the GSH-triggered heterodimer can integrate HA through host-guest interactions to self-assemble into supramolecular nanocomplexes. As a result, the supramolecular nanocomplexes showed excellent HA-mediated tumor targeting by recognizing CD44 overexpressed on the surface of tumor cell membranes. In addition, the biodegradability and bioactivity of HA make it a promising natural biomaterial in nanocarriers for PDT 93. After laser irradiation, the activation of PPa generates ROS and produces antitumor immunogenicity, leading to the infiltration of CTLs into tumor site. More importantly, a combination of PDT-driven immune response and IDO-1 blockade significantly improved the antitumor efficiency compared to PDT treatment alone 93.

In addition to organic nanocarriers, inorganic nanocarriers (e.g., mesoporous silica NPs (MSNs)) are also an important branch to deliver PSs for PDT, as they have many advantages, including tunable size, prone to surface modification, and high colloidal stability 94. Because inorganic nanocarriers are difficult to degrade and easily cause accumulation toxicity, their application is limited 95. Recently, many studies have been devoted to the development of biodegradable inorganic NPs by introducing sensitive bonds; thus, the abovementioned problem has been significantly improved. For example, Xu et al. constructed a GSH-responsive biodegradable multifunctional nanosystem for personalized cancer immunotherapy against local and metastatic tumors 96. The obtained multifunctional nanosystem consisted of MSNs conjugated with neoantigen peptides via disulfide bond 96, in which Ce6 and CpG oligodeoxynucleotide adjuvant were coencapsulated. Upon PET imaging, the multifunctional nanosystem after intravenous administration was effectively accumulated in tumors. Meanwhile, the accumulated Ce6 initiated PDT after laser irradiation, and then PDT-treated tumor sites would recruit numerous DCs to induce ICD 96. Furthermore, the GSH-triggered release of personal neoantigen increased the antigen availability by DCs, which is also a requisite factor for inducing ICD. Therefore, PDT combined with personal neoantigen plus CpG adjuvants achieved synergy, thereby further enhancing the ICD and realizing neoantigen-specific immune response 96.

#### 2.4.3. ROS-Responsive NPs for PDT-driven cancer Immunotherapy

The encapsulation within or conjugation to a ROS-responsive nanocarrier can regulate the release of PSs because tumor cells with higher ROS levels initiate the cleavage of sensitive bonds 97, 98. In addition, the released PSs stimulated by laser irradiation can also produce exogenous ROS. Therefore, the PS-generated ROS combined with intracellular ROS can provide a cascading amplification effect to remotely control the release of cargos 99. For instance, Su et al. designed a multifunctional nanoplatform (NP-sfb/ce6) by encapsulating Ce6 and sorafenib in ROS-responsive polymeric NPs for antitumor immunotherapy (**Figure 5C**) 83. First, they synthesized PEGylated hyperbranched polyphosphates with ROS-responsive capability by inserting thioketal linkers and used them to construct polymeric NPs 83. After laser irradiation, the NP-sfb/ce6 was rapidly disassembled because the PDT-generated singlet oxygen cleaved the thioketal linkers, leading to a cascade amplification to enhance the drug release of sorafenib. In addition, low-dose PDT significantly inhibited the primary tumor growth as well as distant tumor growth by combining with the rapidly released sorafenib, due to synergistically inducing ICD, reversing the immunosuppressive TME and increasing the infiltration of CTLs 83.

#### 2.4.4. Hypoxia -responsive NPs for PDT-driven cancer immunotherapy

Unlike the abovementioned strategies to ameliorate hypoxia TME, application of tumor hypoxia by rapidly transforming large-sized NPs to small-sized nanocomplexes would better regulate the delivery of nanocarriers *in vivo* and accumulation of tumor sites 100, 101. This is because the large sized NPs will undergo long blood circulation, while the small sized NPs can achieve deep tumor penetration or accumulation in specific organs, such as lymph nodes and kidney 102. Recently, a big challenge still exists for cancer vaccines that improve the delivery efficiency of both neoantigens phagocytized by DCs and adjuvant to tumor draining lymph nodes (DLNs) 84. To solve this problem, Kim et al. prepared a Ce6-doped mesoporous silica nanocarrier decorated with a PEGylated azobenzene linker, which was loaded with CpG and glycol chitosan (GC), in which the CpG and GC could form complexes though electrostatic interaction (**Figure 5D**) 84. Once this nanoplatform arrives at a hypoxia TME, the hypoxia-responsive bond (azobenzene linker) will be rapidly cleaved to release the CpG/GC complex. Furthermore, the Ce-6 generated ROS upon laser irradiation not only induced PDT but also enhanced the cleavage of azobenzene linker due to further oxygen consumption by PDT 84. Moreover, the tumor cells after treating with PDT produced neoantigens and subsequently phagocytized by DCs to provide immune response. In addition, they showed that PEGylated NPs successfully escaped from DLNs to accumulate at the tumor site, while dePEGylated NPs reached the DLNs by cleaving the hypoxia-responsive labile linker and then endocytosed by DCs, thereby improving the antigen presentation activity of DCs 84, 103. In another study, Mi et al. designed hypoxia-activatable polymeric micelles to skillfully integrate therapeutic agents into one nanosystem. After arriving at the hypoxia tumor tissue, the nanosystem was selectively activated to release the therapeutic agents. Subsequently, the released agents not only eradicated the primary tumors, but also induced robust systemic immune responses. More importantly, this nanosystem in combination with CpG and aCTLA4 achieved a synergistic effect to inhibit tumor metastasis and recurrence 104.

#### 2.4.5. Multiple-responsive NPs for PDT-driven cancer immunotherapy

In biological systems, the change in the behavior of a biomacromolecule is often a result of its response to a combination of multiple environmental changes 105. To further improve the tumor-targeted drug delivery, Boolean logic operations involving logic-gated devices have drawn increasingly more attention. Based on Boolean logic rules, better logic operations were established by incorporating more input signals (e.g., tumor hallmarks) 106, 107. Inspired by this feature, construction of versatile nanoplatforms which can respond to multiple stimuli of pH/GSH/enzyme would facilitate the on-demand release of therapeutic cargos. For example, Hou et al. designed Boolean logic prodrug NPs (BLPNs) for precise cancer immunotherapy 108. The BLPNs are composed of two biologically stimulant-responsive polymer prodrugs, one of which is conjugated with an immune activator (PPa) and the other is modified to an immune inhibitor (NLG919) by disulfide bond. In addition, both the polymer prodrugs were PEGylated by inserting MMP-2/9-responsive peptide and showed an ultra-pH-sensitive capability because of the presence of the structure of 2-(diisopropylamino) ethyl methacrylate. Based on Boolean logic, a series of BLPNs obtained by adjusting the input combinations of three tumor hallmarks (pH/GSH/enzyme) will yield different YES/AND logic outputs. They found that the BLPNs improved the deep tumor penetration and cellular uptake by MMP-2-mediated dePEGylation. With the progress of the protonation in the endosomal vesicles of cancer cells, the BLPNs would rapidly dissociate to acquire acid-activatable photoactivity, and then produce PDT-induced immune response upon laser irradiation. More importantly, the GSH-triggered drug release of NLG919 controllably reversed the immunosuppressive TME by inhibiting the IDO pathway. As a result, this multiple-responsive nanoplatform achieved precise cancer immunotherapy by using Boolean logic operations 108.

### 2.5. Subcellular targeted NPs for PDT-driven cancer immunotherapy

So far, several smart nanotechnology formulations have been developed to deliver PSs into tumor tissue by utilizing the EPR effect. Upon a local laser irradiation, the PS-generated ROS damage adjacent biomolecules, thereby inducing PDT 109, 110. However, ROS (e.g., ^1^O_2_) has a short lifespan, significantly limiting their diffusion in cytoplasm as well as among tumor cells, causing a discounted PDT efficacy 111, 112. Therefore, precise delivery of PSs to ROS-related subcellular organelles (including ER 113, mitochondrial 114, lysosome 115, and cell nucleus 116) as well as plasma membrane (PM) 117 effectively avoided the fast decay of cytoplasm-localized PDT (cPDT)-derived ROS, leading to a maximal antitumor effect of PDT.

**Figure 6:**
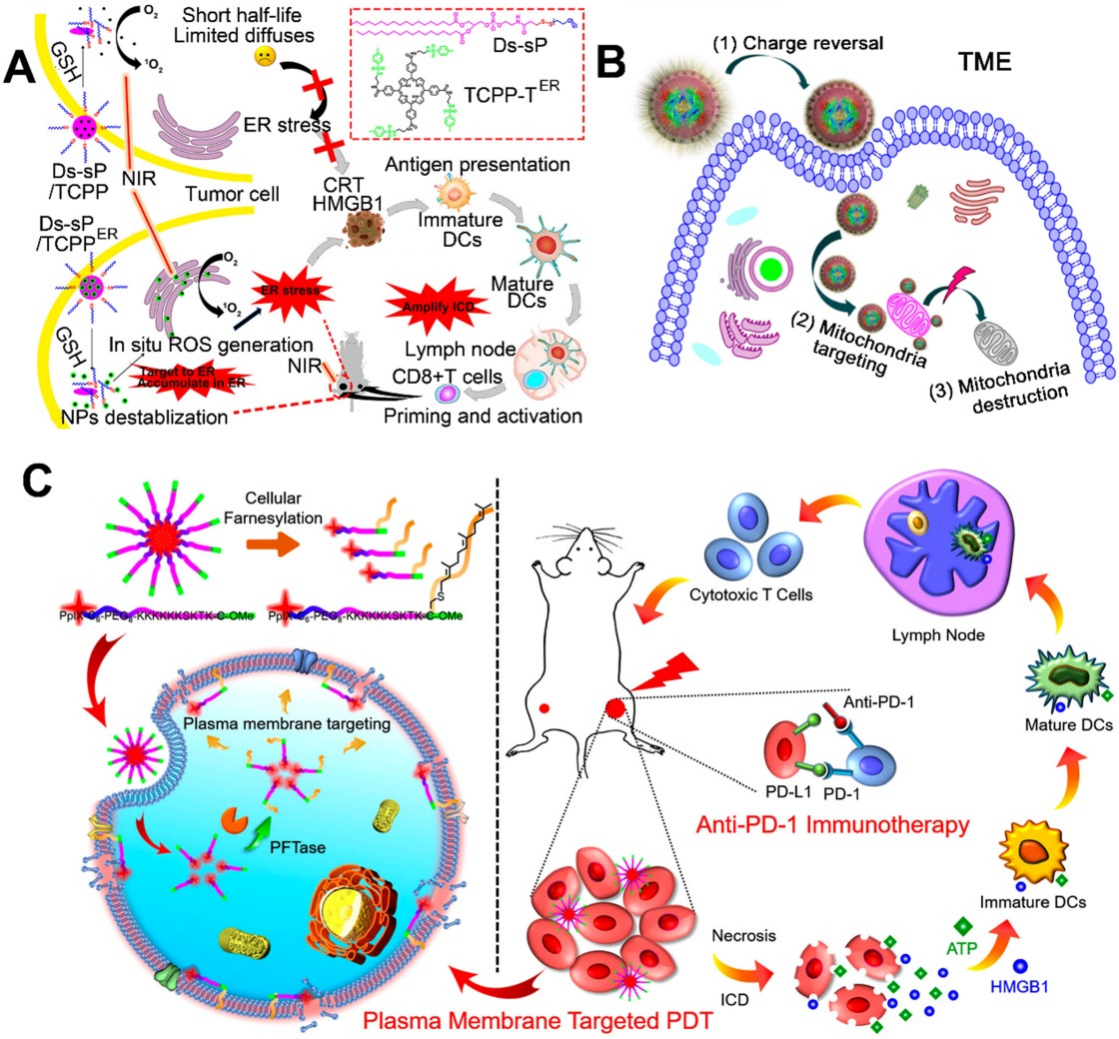
Schematic illustration of various subcellular targeted NPs for improved PDT-driven cancer immunotherapy. (A) ER-targeting Ds-sP/TCPP-T^ER^ NPs, (B) Ce6-loaded mitochondrial-targeting NPs, (C) enzyme-driven PM-targeting PCPK NPs. Reproduced with permission 118, 87, 120. Copyright 2020, American Chemical Society; Copyright 2018, American Chemical Society; Copyright 2019, American Chemical Society.

ER in the cell plays a role in the synthesis and transport of protein, intracellular calcium storage, and the regulation of intracellular calcium levels to maintain its balance. Cell apoptosis mediated by ER pathway has become a new field of research on apoptosis 121. As mentioned above, ROS-mediated PDT induced ICD by causing sequential changes including, but not limited to, CRT exposure and HMGB1 release to produce immune response 34, 122. Recently, several studies showed that the CRT is located in ER, so that the ROS-induced ER stress provided a high-efficiency boost to CRT exposure to evoke a strong ICD 123-125. For example, Deng et al. synthesized a ER-targeted PS (TCPP-T^ER^) using *N*-tosylethylenediamine as the PS (TCPP) and a reduction-sensitive polymeric conjugate (Ds-sP) (**Figure 6A**) 118. Subsequently, the synthesized Ds-sPs simply self-assembled into NPs, with TCPP-T^ER^ coencapsulated into the nanostructures. In the presence of GSH, the self-assembled Ds-sP/TCPP-T^ER^ was rapidly destabilized and enhanced the release of TCPP-T^ER^, because GSH triggers the cleavage of reduction-sensitive linkers. Compared to TCPP-T, the released TCPP-T^ER^ from Ds-sP/TCPP-T^ER^ by using *N*-tosylethylenediamine showed an excellent ER targeting capability and *in situ* ROS generation, leading to an enhanced ER stress 118. Furthermore, they found that Ds-sP/TCPP-T^ER^ under laser irradiation induced more CRT exposure on the membrane of tumor cells in comparison with Ds-sP/TCPP-T, providing amplified ICD, thus resulting in the enhanced infiltration of CTLs.

In addition to ER, another ROS-sensitive subcellular organelle, mitochondria, has drawn much attention in increasing the efficiency of PDT 126-128. As the cells' powerhouse, the mitochondria are tightly linked to many intracellular activities, including programmed cell death (apoptosis), ROS generation, and ATP production 129, 130. Furthermore, cell apoptosis and release of ATP are associated with ICD. Therefore, delivery of PSs to mitochondria using a reasonable nanosystem could be a potential strategy for enhancing cancer immunotherapy 87, 119. For example, Liu et al. designed mitochondria-targeting NPs loaded with Ce6 to improve PDT-mediated immunotherapy, in which a triphenylphosphonium (TPP)-modified cationic hollow silica precursor acted as nanocarriers (**Figure 6B**) 87. Subsequently, the positively charged core coated with a negatively charged pH-responsive PEG layer by electrostatic interaction acted as a protective shell 87. Thanks to the negatively charged pH-responsive PEG layer, the mitochondria-targeting micelles with a positive charge avoided rapid clearance during the circulation, thus leading to an obvious tumor accumulation. After reaching the acidic TME, the pH-responsive PEG layer was rapidly decomposed and exposed the TPP molecules, thus increasing the cellular uptake of positively charged micelles and recovering the targetability to the mitochondria. More importantly, the Ce6-loaded micelles upon laser irradiation induced improved immune responses via ROS generation in mitochondria 87, 119.

Compared with conventional cPDT-induced apoptosis, necrosis provides a more immunogenic result from the accelerated release of DAMPs 131, 132. PM-targeted PDT led to PM rupture by causing a series of changes (e.g., enhanced membrane permeability and lipid peroxidation) 133. Subsequently, PM rupture induced cell necrosis by rapidly releasing the intracellular contents, thus leading to a strong antitumor immune response 120. For instance, Zhang et al. constructed enzyme-driven PM-targeted NPs (PCPK NPs) self-assembled from chimeric peptide (PpIX-C_6_-PEG_8_-KKKKKKSKTKC-OMe, termed as PCPK) (**Figure 6C**) 120. The self-assembled PCPK NPs acquired the PM targeting ability through the protein farnesyltransferase (PFTase)-mediated enzymatic conversion, and then the obtained farnesylated product was tightly anchored on the PM 134-136. A After laser irradiation, the PCPK produced PM-localized ROS and subsequently induced an intense PDT efficacy, resulting in lipid peroxidation and PM rupture. This localized PDT-driven PM damage induced an obvious cell necrosis to rapidly release ATP and HMGB1, leading to a strong immune response 120. More importantly, this PM-PDT strategy in combination with checkpoint blockade therapy effectively inhibited the growth of metastatic tumor.

### 2.6. Carrier-free and small-molecule prodrug-based self-assembled NPs for PDT-driven cancer immunotherapy

**Figure 7:**
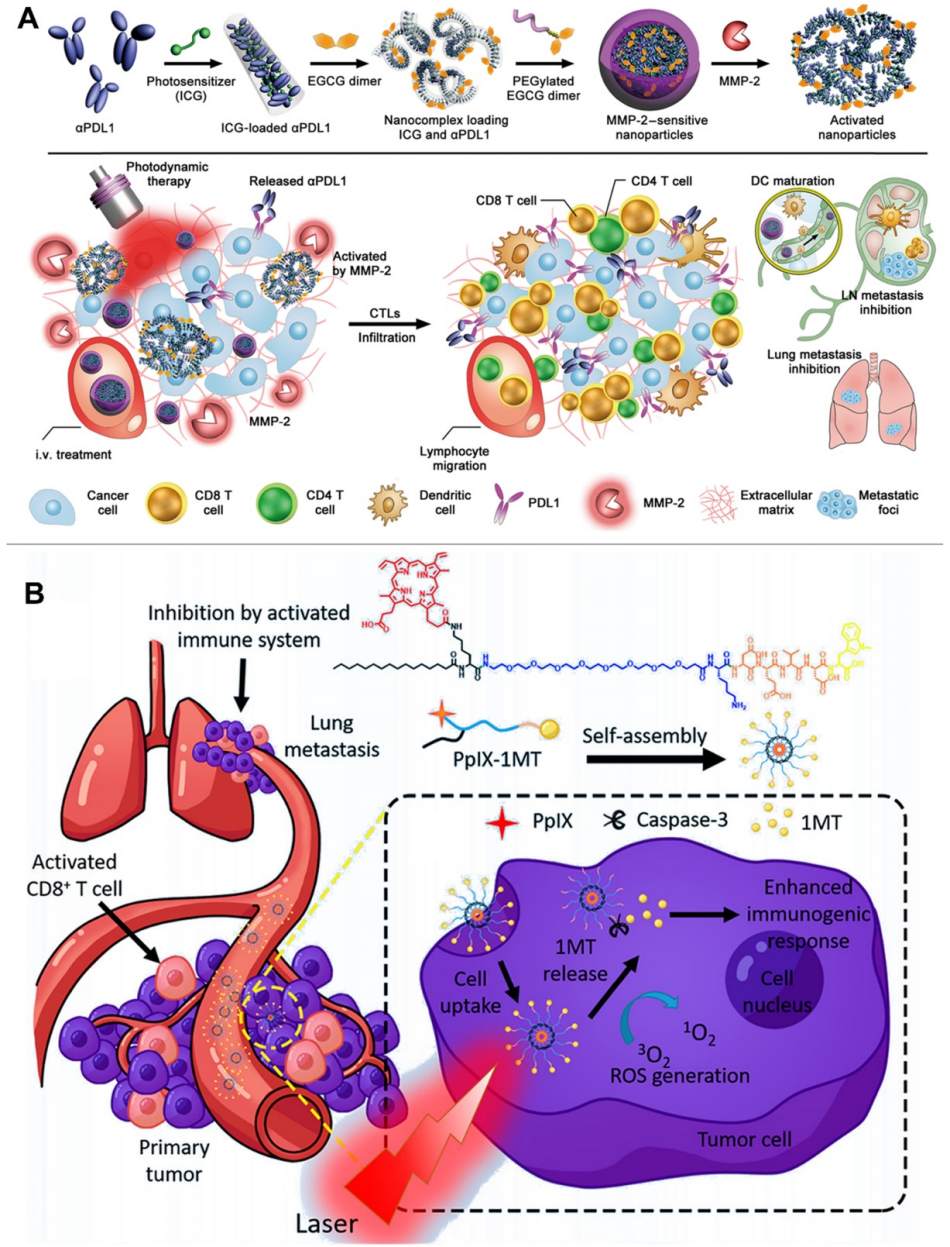
(A) Schematic illustration of preparation of the PEG-coated co-assembled NPs of ICG and αPD-L1, *in vivo* local activation of cancer immunotherapy. Reproduced with permission 139. Copyright 2019, AAAS. (B) Schematic illustration of preparation of small molecule prodrug self-assembled NPs for *in situ* PDT, increased immunogenic response, cascade release of IDO inhibitor, inhibition of lung metastasis. Reproduced with permission 90. Copyright 2018, American Chemical Society.

Different from multifunctional polymeric nanocarriers requiring complex preparation technologies and potential toxic excipients, carrier-free nanoplatforms could be self-assembled from pure therapeutic drugs for delivering PSs without causing side effects from using additional excipients 137, 138. For instance, Wang et al. constructed a TME-activatable carrier-free nanoplatform composed of ICG and αPD-L1 for combination immunotherapy (**Figure 7A**) 139. They showed that the ICG and αPD-L1 could be simply self-assembled into NPs via electrostatic interactions and hydrophobic force, exhibiting a diameter of approximately 14.5 nm in size. Moreover, the self-assembled NPs modified with the MMP-2-liable PEG corona (carrier-free nanoplatform) led to a prolonged blood circulation and thus prevented the compressed αPD-L1 from interacting with normal organs 139. In addition, the carrier-free nanoplatform could be selectively triggered to release ICG and αPD-L1 by utilizing the tumor overexpressed MMP-2, thereby significantly enhancing the accuracy of αPD-L1-based checkpoint blockade therapy. More importantly, the controllable release of ICG produced local ROS to induce strong immune response, which further cooperates with αPD-L1 to achieve high-efficiency cancer immunotherapy for inhibiting tumor growth and metastasis 139. To improve the accuracy of image-guided PDT, Xu et al. designed multifunctional nanocomplexes for a combination delivery of Ce6 and immunoglobulin G (e.g., αCTLA-4 and αPD-L1) 140. The authors revealed that the Ce6 showed a higher affinity to immunoglobulin G compared with previous reported HSA. Furthermore, immunoglobulin G and Ce6 could be self-assembled into stable NPs (chloringlobulin) with the addition of polyvinylpyrrolidone, significantly improving the intratumoral concentration of Ce6 without affecting its metabolic rate in blood 140. Compared with the traditional strategies to deliver PSs, this “immunoglobulin G hitchhiking” approach improved the tumor accumulation of Ce6 and then induced strong immune response to confront the limited clinical treatment effect of checkpoint blockade therapy due to low tumor immunogenicity 140. Therefore, the αPD-L1-based chloringlobulin achieved a cooperative therapy by the integration of PD-L1 blockade therapy, Ce6-mediated PDT, and fluorescence image-guided surgery. More importantly, the authors showed that the dual checkpoint blockade (αCTLA-4 and αPD-L1) therapy in combination with the PDT-induced ICD further enhanced the cancer immunotherapy 140, 141.

Besides the carrier-free self-assembled NPs, the small-molecule prodrug-based self-assembled NPs also have a great potential in clinical translation for anticancer therapy owing to their high drug delivery ability and avoidance of using toxic excipients 142. For example, Zhang et al. constructed a novel small-molecule prodrug (PpIX-1MT) by conjugating the checkpoint inhibitor (1 MT) to PS PpIX by inserting a caspase-responsive chimeric peptide, and then the synthetic PpIX-1MT was self-assembled into NPs (**Figure 7B**) 90. After reaching the tumor site, the PpIX-1MT NPs under laser irradiation generated ^1^O_2_ to cause cancer cell apoptosis, which further activated the overexpression of caspase-3 90. The overexpressed caspase-3 in turn rapidly cleaved the caspase-responsive linker to enhance the release of PpIX and 1MT. The released PpIX enhanced the production of ^1^O_2_ to induce a strong immune response, and the synchronously released 1-MT achieved a cascaded synergistic effect in cancer immunotherapy by relieving the immunosuppressive TME 90.

## 3. Emerging nanomedicines for multi-factor-driven cancer immunotherapy

Although PDT can induce immune response by ICD in certain tumors, its effect is significantly hindered by the complex immunosuppressive TME 143. Therefore, monotherapy based on PDT usually exhibits a limited efficacy to induce antitumor immunity. Recently, several therapeutic strategies also can initiate ICD by directly killing tumor cells, including radiotherapy, chemotherapy, and photothermal therapy (PTT) 34, 35. Combination of multiple therapies might achieve a synergistic effect to increase the intensity of immune response. Nanomedicine-based delivery can coencapsulate different drugs into one nanoplatform and alleviate the side effect due to nonspecific systemic distribution. We discuss emerging nanomedicines for improving the immune response by combination strategies such as PDT/ chemotherapy and PDT/PTT. More importantly, the combination strategies plus checkpoint blockade immunotherapy will achieve a durable and efficient antitumor treatment effect.

### 3.1. Emerging nanomedicines for PDT-chemo-driven cancer immunotherapy

**Figure 8:**
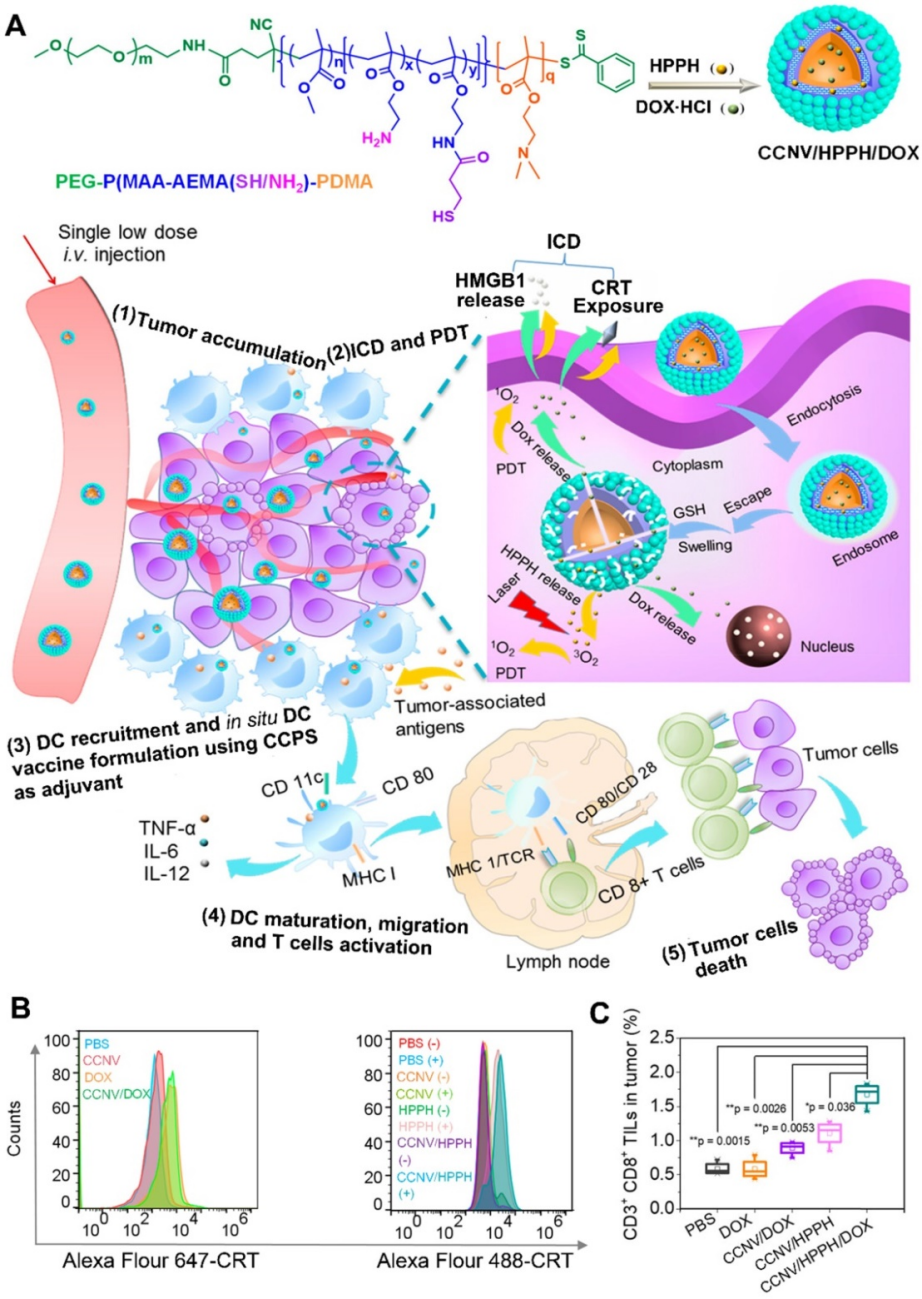
(A) Schematic illustration of preparation of CCNV/DOX/HPPH as *in Situ* DC Vaccine for synergistically inducing ICD by chemotherapy and PDT, CTL activation and vaccine immunotherapy. (B) Flow cytometry characterization of CRT exposure by measuring various formulations stained with specific fluorescent probe of CRT. (C) Frequency of CD3+CD8+ TILs in tumors treated with different formulations. Reproduced with permission 144. Copyright 2019, American Chemical Society.

For a long time, chemotherapy has been the mainstay of cancer treatment. Hence, chemotherapy combined with other therapies always draw much more attention 18. Some chemotherapeutic agents (e.g., doxorubicin) can trigger ICD to induce systemic immune response 34. Chemotherapy-mediated ICD facilitates the release of danger signals (e.g., CRT) from dying tumor cells, which is similar to PDT-mediated ICD, and then achieve a synergistic effect to enhance immune activation 18. For example, Chen et al. developed chimeric cross-linked nanovesicles (CCNVs) self-assembled from a triblock copolymer, which both acted as nanocarriers and adjuvants (**Figure 8**) 144. With the conjugation of protonatable groups, the CCNVs simply escaped from the endosome after cellular internalization. In addition, the cross-linked structure via thiol groups not only helped to avoid drug leakage, but also regulated the release of loaded cargos in response to GSH. After the coencapsulation of HPPH and DOX, the CCNV/DOX/HPPH under laser irradiation induced ICD by both DOX and HPPH-mediated PDT, which would achieve a combinational effect to improve DC recruitment and TAA secretion in tumor site 144. Furthermore, the embedded amines of CCNV effectively enhanced antigen presentation and DC maturation by acting as an adjuvant. As a result, this smart *in situ* DC vaccine strategy induces an intense immune response by the integration of chemotherapy, PDT, and immunotherapy 144. Furthermore, Yang et al. designed cascade chemo-PDT by using a ROS-responsive nanoplatform (^TK^HNP-C/D) composed of a polymer-lipid hybrid nanocarrier as well as the coencapsulation of DOX and Ce6 to provide the checkpoint blockade immunotherapy 145. In their study, the hybrid nanocarrier consisted of a hydrophilic shell and a hydrophobic core: The hydrophilic shell includes DSPE-PEG and neutral lipid lecithin to increase the stability and drug loading efficiency of nanocarriers; the hydrophobic core contains a ROS-responsive poly(thioketal phosphoester) to regulate the release of loaded cargos 145. They found that the ^TK^HNP-C/D upon laser irradiation cleaved the ROS-sensitive groups by the Ce6-generated ROS and in turn accelerated the release of Ce6 and DOX, leading to a self-amplified ROS-induced PDT. In addition, the released DOX combined with the Ce6-generated ROS achieved a cascade chemo-PDT to trigger a strong antitumor immune response by facilitating DC recruitment and CTL infiltration to tumor site. More importantly, the aPD-L1 in combination with ^TK^HNP-C/D plus laser irradiation generated an immune memory effect to inhibit distant tumor growth 145.

Apart from DOX, oxaliplatin (OXA) causes the release of DAMPs to effectively induce ICD, leading to a strong immunity response 146. For example, He et al. developed a core-shell nanoplatform to improve checkpoint blockade immunotherapy, in which the shell included pyropheophorbide-lipid conjugates (pyrolipid) to induce PDT, and the core contained oxaliplatin prodrugs to produce chemotherapy 146. The authors found that the synthetic OXA prodrug and pyrolipid of this core-shell nanoplatform not only enhanced the drug loading capability of parent drugs (oxaliplatin and pyropheophorbide-a), but also prolonged their blood circulation 146. Moreover, the pyrolipid-induced PDT synergized with OXA-elicited chemotherapy to promote CRT exposure and antigen presentation, resulting in tumor-specific immune responses. Furthermore, this synergized chemo-PDT plus αPD-L1 therapy effectively inhibited the metastatic metastasis of colorectal cancer. Besides the checkpoint blockade of PD-L1, the CD47 blockade has been utilized in clinical trials 147-149. For instance, Zhou et al. constructed TME-responsive prodrug vesicles in combination with CD47 blockade for cancer immunotherapy 150. In this study, prodrug vesicles underwent a charge transformation at tumor tissue by the MMP-2-triggered cleavage of PEG-PS conjugates, which not only enhanced the cellular uptake and deep tumor penetration, but also selectively released the PS. After internalization by tumor cells, the prodrug vesicles rapidly released OXA in response to GSH. Furthermore, the released OXA and PS plus laser irradiation reached a synergic chemo-PDT to induce ICD, and further utilized CD47 blockade to relieve immunosuppression, leading to a combinational strategy for cancer immunotherapy 150. To further enhance the drug delivery efficacy, Feng et al. synthesized a GSH-responsive heterodimer of NLG919 and PPa, which could be simply self-assembled into NPs due to the insertion of disulfide bond, resulting in an ultrahigh drug loading 151. To improve the stability and tumor accumulation of NPs, a thioketal bond-linked PEGylated OXA prodrug served as a protective shell. After the first wave light irradiation, the PEGylated NPs produced numerous PPa-mediated ROS, which triggered dePEGylation by the cleavage of thioketal bond, leading to deep tumor penetration. After internalization by tumor cells, the dePEGylated NPs not only facilitated the release of NLG919 and PPa via the GSH-induced degradation of heterodimer, but also increased the OXA release form prodrug. After second light irradiation, the released OXA and PPa achieved a synergetic chemo-PDT to induce immune response. Furthermore, the GSH-mediated NLG919 release suppressed IDO-1 activity to relieve immunosuppressive TME 151.

**Figure 9:**
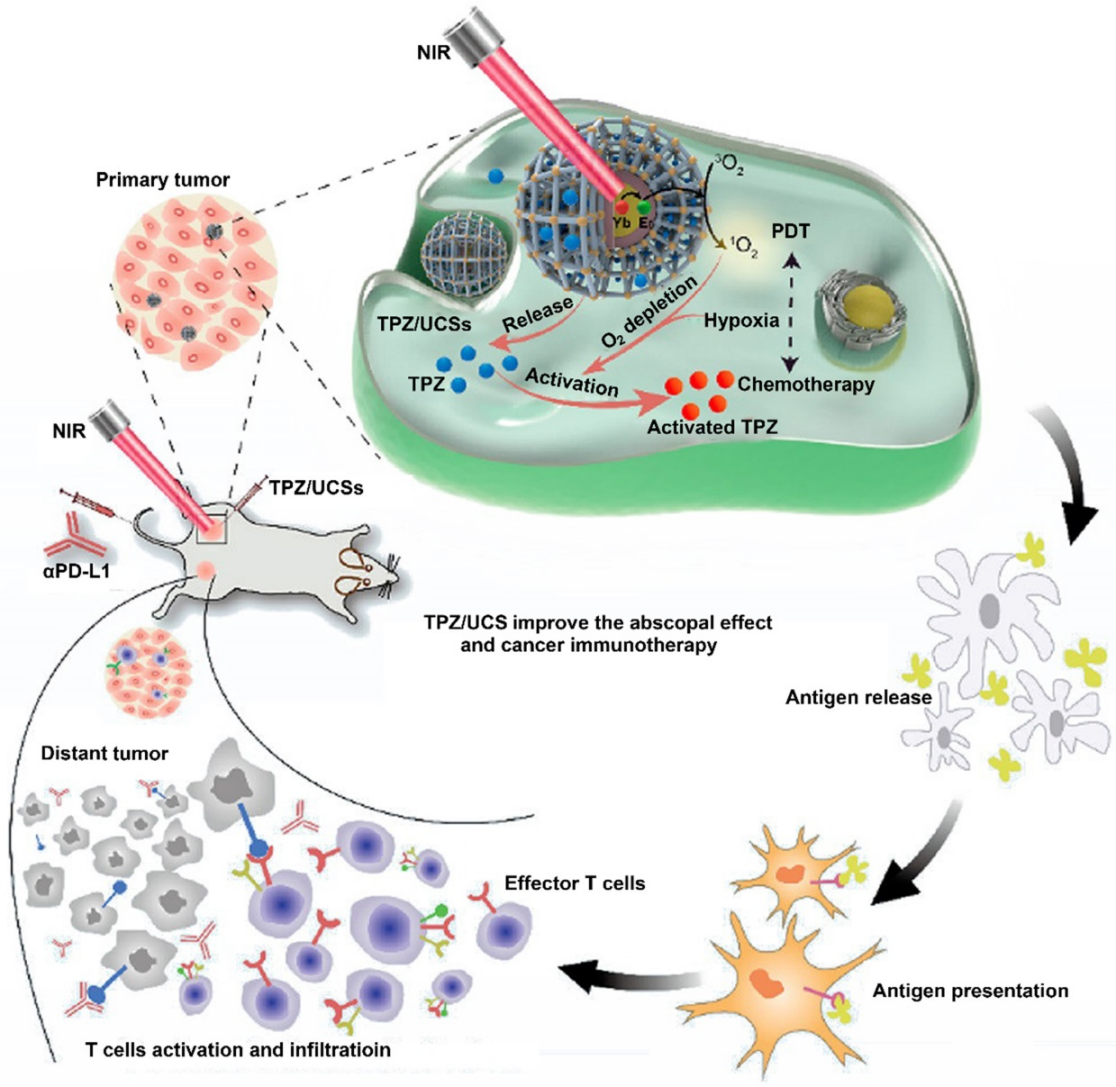
Schematic illustration of UCN@MOF nanostructures loaded with TPZ for cancer treatment by integration of hypoxia-activated chemotherapy, NIR light-triggered PDT and immunotherapy. Reproduced with permission 157. Copyright 2020, American Chemical Society.

PDT itself involves continuous oxygen consumption, which would further aggravate tumor hypoxia 152-154. Recently, studies constructed hypoxia-activated prodrug (HAP) nanoplatforms instead of aforementioned hypoxia relief strategies to enhance anticancer treatment 155-157. For example, Shao et al. developed tirapazamine (TPZ)-loaded UCN@MOFs (TPZ/UCSs) to achieve combinational chemo-PDT (**Figure 9**) 157. With the special heterostructure, UCSs absorbed long-wavelength light (LWL), and further transferred energy form UCNs to PS, resulting in a deeper-penetrating PDT. In this study, the PS-based MOF has a high PS loading ability, and the well-dispersed PSs effectively avoided self-quenching to induce PDT 157. Furthermore, the loaded TPZ, a hypoxia-activated prodrug, was efficiently activated by the PDT-aggravated tumor hypoxia. Subsequently, the activated TPZ produced toxic oxidizing radical species, which further synergized with the Ce6-generated ROS to induce a strong antitumor immune response. More importantly, the integration of αPD-L1 with TPZ/UCSs produced specific tumor infiltration of CTL to suppress distant tumors through this combinational strategy of immunotherapy, hypoxia-activated chemotherapy, and LWL-mediated PDT 157.

### 3.2. Emerging nanomedicines for PDT-PTT-driven cancer immunotherapy

Using the photo-absorbing agents, PTT achieved targeted-tissue-specific therapy via converting light into heat and synergized with the oxygen-dependent PDT against hypoxic tumor. In addition, this localized thermal ablation effect also induced ICD, leading to the desired antitumor immune responses 158-160. So far, many photothermal agents (PTAs) have been reported, such as inorganic metals, transition metals, magnetic materials, organic dyes, organic polymers, and organic semiconductor 57, 161-165.

**Figure 10:**
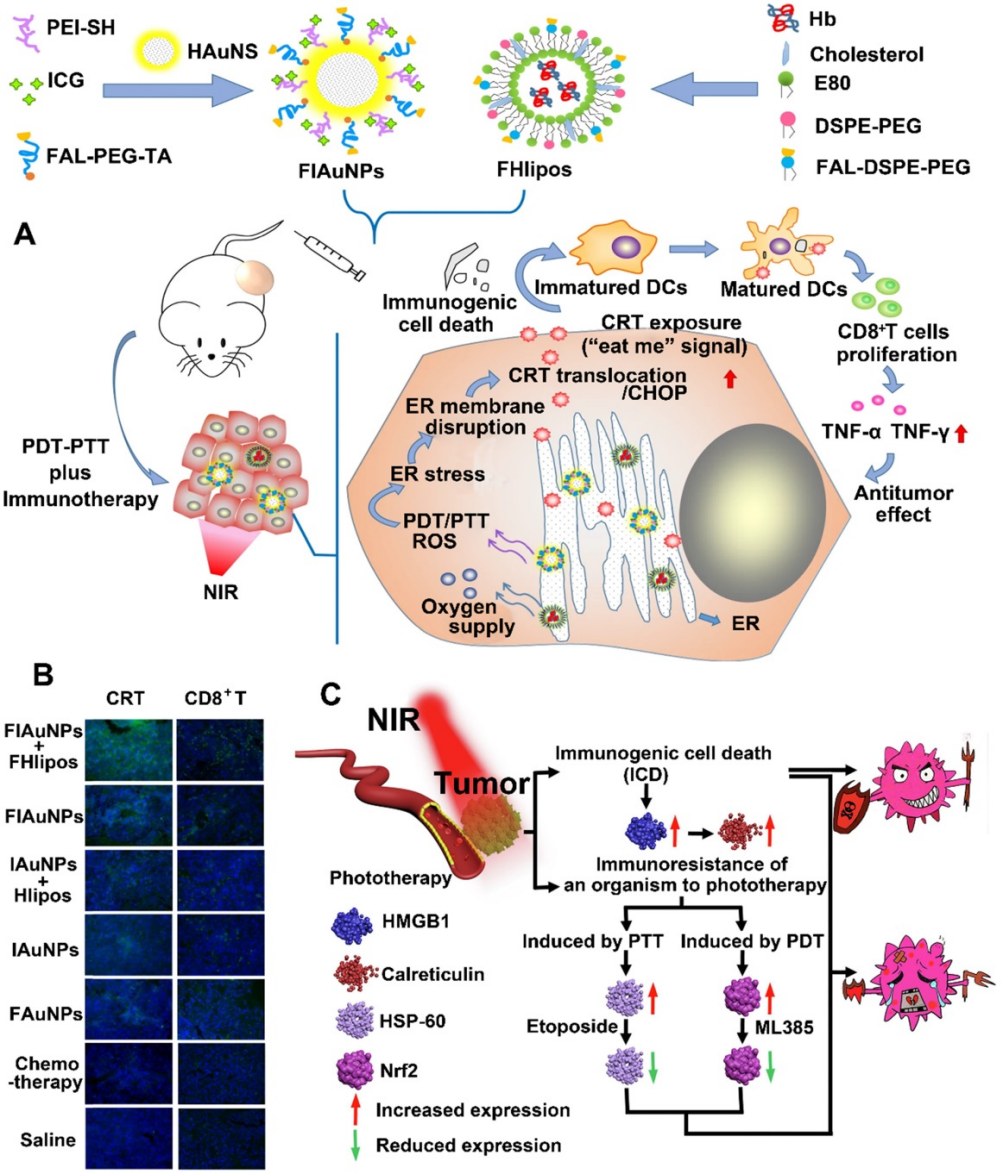
(A) Schematic illustration of preparation of ER-targeting and ER-targeting FIAuNPs and FHlipos for combinational therapeutic strategies including ER-targeting PTT, ER-targeting PDT, and synergistically inducing ICD by PTT and PDT. (B) CLSM imaging of tumor sections treated with various formulation for detecting immunofluorescent staining of CD8^+^ T cells and CRT expression. Reproduced with permission 57. Copyright 2019, Nature publisher. (C) Schematic illustration of improved PTT/PDT-driven cancer immunotherapy by combination with both HSP60 and NRF2 to inhibit immune-resistance caused by PTT and PDT. Reproduced with permission 162. Copyright 2020, Elsevier Ltd.

Inorganic PTAs have many advantages in PTT-mediated cancer treatment, including strong NIR responsiveness, resistance to photodegradation, and high photothermal conversion efficiency. Therefore, inorganic PTAs are an excellent choice to induce strong immune responses. For example, You et al. designed a dual ER-targeting nanosystem composed of ICG-grafted hollow gold NPs (FIAuNPs) modified with pardaxin (FAL) peptides and FAL-modified O_2_-carrying Hb liposomes (FHlipos) to achieve a synergistic PDT-PTT-induced immunotherapy (**Figure 10A**) 57. Thanks to the modification of FAL peptide, this nanosystem realized ER-targeted accumulation and direct ROS-based ER stress, leading to ER-localized PDT. To relieve tumor hypoxia, the FHlipos released O_2_ from Hb, further enhancing the efficiency of ER-localized PDT 57. In addition, this FIAuNPs under laser irradiation resulted in tumor hyperthermia (PTT) due to the special structure of ICG-grafted hollow gold NPs, which effectively converted light into heat 57. Furthermore, they showed that the integration of ER-targeting PDT-PTT caused much more CRT exposure, DC maturation, and proliferation of CTLs compared with nontargeting nanosystem, leading to a promoted ICD-associated immunological response (**Figure 10B**) 57. To further enhance the effect of PDT and PTT, Chang et al. modified Cu_2_MoS_4_ (CMS) nanosheets with the deposition of plasmonic gold NPs to develop novel CMS/Au heterostructures, which showed a widened optical absorption band based on orbital hybridization 161. Compared to single CMS, the CMS/Au heterostructures triggered the reaction of H_2_O_2_ to produce O_2_ by acting as catalysts and further improved the effect of PDT by relieving tumor hypoxia. Based on these advantages, they found that PDT-PTT induced by CMS/Au heterostructures enhanced cytokine secretion and DC maturation to elicit immune responses and produce a memory effect of CTLs to suppress tumor metastasis.

Unlike metal PTAs (e.g., Au), some nonstoichiometric materials (e.g., CoWO_4_-x) under a single wavelength light irradiation showed both PDT and PTT properties through the synchronous generation of ROS as well as hyperthermia 162. In addition, these biomaterials have multimodal imaging abilities due to the presence of tungsten elements, which make them excellent PA agents 162. Taking these advantages, Liu et al. designed a multifunctional CoWO_4_-x nanoplatform to enhance the PDT/PTT immunotherapy, in which the surface of CoWO_4_-x NPs was modified with hydrophilic polymers to improve the biocompatibility and stability (**Figure 10C**) 162. With tungsten elements and NIR absorption properties, CoWO_4_-x NPs showed excellent PA and CT imaging, which helped to better monitor the *in vivo* dynamics of NPs. Furthermore, the nonstoichiometric CoWO_4_-x NPs under laser irradiation induced both PDT and PTT, resulting in increased HMGB1 expression and CRT exposure. However, the ICD induced by phototherapy (PDT-PTT) provided a disappointing therapeutic effect. They found that phototherapy induced the overexpression of immunosuppressive agents (NRF2 and HSP60). Therefore, CoWO_4_-x nanoplatform combined with specific inhibitors of immunosuppressive agents (ML385 and etoposide) realized a synergistic therapeutic effect of phototherapy and immunotherapy 162. Recently, organic semiconducting polymer nanoplatforms (OSPNs) have drawn much more attention. This is because the OSPNs not only induce PDT and PTT by converting optical energy into ROS and hyperthermia, but also exhibit some unique advantages, including excellent biocompatibility, good photostability, tunable optical properties, and large absorption coefficients 166-168. For example, Pu et al. developed a prodrug-based OSPN consisting of NLG919-conjugated semiconducting polymer NPs via ^1^O_2_ cleavable linkers to achieve an accurate phototherapy-induced immunotherapy 164, 169. This OSPN under laser irradiation induced synergistic PDT and PTT by producing ^1^O_2_ and hyperthermia to ablate tumors. Subsequently, the generated ^1^O_2_ selectively cleaved the ^1^O_2_-responsive linkers to accelerate the release of NLG919. More importantly, the phototherapy induced ICD to significantly enhance the proliferation of CTLs, and then the locally released NLG919 restored the activation of CTLs by inhibiting the IDO pathway. As a result, the OSPN achieved synergistic antitumor immunotherapy agonist malignant tumors 164. Bioinspired polydopamine (PDA) prepared by the autooxidation polymerization of dopamine has many properties, including excellent photothermal conversion efficiency, strong adhesive property, and good biocompatibility 170. Thanks to these properties, Yan et al. designed PDA-coated UCNs loaded with Ce6 to combine PDT and PTT to induce immunotherapy against disseminated tumors 165. Compared to single approach (PDT or PTT), they found that the synergistic phototherapy promoted TAA release and DC maturation, inducing a strong immune response. Moreover, this synergistic phototherapy also recruited macrophages and B cells, eliciting an innate immune response. Furthermore, this nanoplatform in combination with αPD-1 activated the proliferation of memory T cells to prevent tumor from relapse and metastasis 165.

## 4. Conclusions and future perspectives

Although cancer immunotherapy has achieved numerous clinical success, patients with cancer have a low response rate. Progressive nanomedicines significantly improve the efficacy of PDT by overcoming the main challenges, including ACQ, tumor hypoxia, and off-target effects. As a result, the facilitated PDT significantly increase the potential to induce immune responses, thereby further driving systemic cancer immunotherapy. We reviewed several emerging nanomedicines in the skillful incorporation of PDT with immunotherapy to better understand the intrinsic advantages of combinatorial therapy. Moreover, nanoparticle-based conventional cancer treatments (e.g., PTT and chemotherapy) can also induce ICD via sequential changes, achieving desirable synergistic effects in combination with PDT to initiate intense immune responses.

Despite the development of nanomedicines in PDT-driven cancer immunotherapy, there are still several challenges and opportunities in the following areas. First, the effective immune response for immunotherapy urgently needs the simultaneous activation of multiple immune signaling pathways 77. Therefore, it still requires advanced biomaterials to optimize multifunctional noncarriers for the codelivery of various immunomodulators to specific cells. Second, components of polymer-based nanomedicines and inorganic nanomaterials usually exhibit poor biodegradability, significantly limiting their clinical translation 171, 172. Hence, small molecule based self-assembled nanomedicines with well-defined chemical structures are suitable for clinical translation 173-175. Third, new strategies (e.g., ACT) combined with PDT can provide the opportunity to enhance the immune response by engineering immune cells. Moreover, nanosized extracellular vesicles derived from immune cells can serve as excellent biocompatible carriers with good tumor-targeting ability 77, 173. Fourth, ferroptosis, a nonapoptotic programmed cell death modality, not only supplies O_2_ to increase the efficacy of PDT, but also produces ROS to induce immune responses 176. More importantly, based on their intrinsic connections of O_2_ and ROS, a combination of PDT and ferroptosis will achieve a synergistic effect to initiate strong immune response. In addition, PDT can activate T lymphocytes to release IFN-γ, which will facilitate the ferroptosis-related lipid peroxidation to further improve the ICD effect 177-180. Therefore, novel nanoplatforms developed by the rational integration of PDT, ferroptosis, and immunotherapy have attracted much attention for cancer treatment 180. Finally, in-depth exploration of the relationship between PDT and cancer immunotherapy is still needed, and more advanced nanomedicines will boost the effectiveness and safety of combinational cancer immunotherapy 181.

## Figures and Tables

**Table 1:** Summary of emerging tumor hypoxia-reversed nanomedicines for PDT-driven cancer immunotherapy. (↑= upregulation, ↓= downregulation).

Formulations	Therapeutic agents	Immunologic modulation	Ref
AuNC@MnO_2_ NPs	AuNCs, MnO_2_	TAAs, mDC, CD4^+^, CD8^+^, NK↑	41
Mn@CaCO_3_/ICG@siRNA NPs	ICG, MnO_2_, siRNA	TAAs, mDC, IL12, IL18, CD4^+^, CD8^+^, INF-γ ↑ PD-L1 ↓	42
Phototherapy hydrogel	Ce6, CAT	TAAs, mDC, CD4^+^, CD8^+^↑ HIF-1α, VEGF↓	52
R837-loaded PLGA NPs	Ce6, CAT, α-CTLA4	TAAs, mDC, CD4+, CD8+↑CTLA-4↓	54
HSA/Hb NPs	Ce6	TAAs, mDC, CD4^+^, CD8^+^, NK↑	43
ICG modified gold nanospheres	ICG,	TAAs, mDC, CD4^+^, CD8^+^↑	57
Fluorinated polymer NPS	Ce6, NLG919	TAAs, mDC, CD4^+^, CD8^+^, Trp↑ Kyn↓	44
DEX-HAase NPs	Ce6, HAase, α-PD-L1	TAAs, mDC, CTLs, INF-γ, M1 macrophage↑ M2, PD-L1 ↓	45
HPR@CCP NPs	Ce6, HAase, Cas9-Ptpn2 plasmids	TAAs, mDC, CD^+^8, INF-γ, TNF-α↑	62
PM-IR780-Met NPs	IR780, Met	TAAs, mDC, CTLs↑ Tregs, MDSC↓	46
IR775@Met@Liposomes	IR775, Met	TAAs, mDC, CTLs, INF-γ↑, PD-L1↓	63

**Table 2:** Summary of emerging TME-responsive nanomedicines for PDT-driven cancer immunotherapy. (↑= upregulation, ↓= downregulation).

Formulation	Therapeutic agents	Stimuli	Immunologic modulation	Ref
PDPA-PPa micelles	PPa, SiRNA	pH	TAAs, mDC, HSP70, CD8^+^, NF-κB↑ PD-L1↓	86
pRNVs/HPPH/IND nanovesicles	HPPH, pRNVs, IND	pH	TAAs, DC, mTOR, CD8^+^ ↑ Treg↓	36
porphyrin-phospholipid liposomes	PPa, NLG-8189	GSH	TAAs, mDC, CD8^+^, INF-γ, Trp↑ Kyn↓	82
supramolecular nanocomplexes	PPa, NLG919	GSH	TAAs, mDC, CD8^+^, Trp↑ Treg, Kyn↓	93
biodegradable inorganic NPs	Ce6, CpG	GSH	TAAs, mDC, CD8^+^↑	96
NP-sfb/Ce6 NPs	Ce6, sorafenib	ROS	TAAs, mDC, CD8^+^↑ MDSCs↓	83
Ce6-doped mesoporous silica NPs	Ce6, CpG	Hypoxia	TAAs, mDC, CD4^+^, CD8^+^↑	84
hypoxia-activatable polymeric micelles	DOX, ICG, CpG, αCTLA4	Hypoxia	TAAs, mDC, CD4^+^, CD8^+^ TNF-α, IFN-γ, IL-12p70↑	104
Boolean logic prodrug NPs	PPa, NLG919	pH/GSH/MMP-2	TAAs, mDC, CTLs, Trp↑ Treg, Kyn↓	108
